# Butenolide Derivatives with α-Glucosidase Inhibitions from the Deep-Sea-Derived Fungus *Aspergillus terreus* YPGA10

**DOI:** 10.3390/md17060332

**Published:** 2019-06-03

**Authors:** Zhongbin Cheng, Yuanli Li, Wan Liu, Lijun Liu, Jie Liu, Wangjun Yuan, Zhuhua Luo, Wei Xu, Qin Li

**Affiliations:** 1 Pharmaceutical College, Henan University, Kaifeng 475004, China; czb360@126.com (Z.C.); lyl328743993@163.com (Y.L.); 18737806806@163.com (W.L.); 15736871748@163.com (L.L.); ll18737801136@163.com (J.L.); 2 Key Laboratory of Marine Biogenetic Resources, Third Institute of Oceanography, Ministry of Natural Resources, Xiamen 361005, China; luozhuh@tio.org.cn; 3 Eucommia Ulmoides Cultivation and Utilization of Henan Engineering Laboratory, Kaifeng 475004, China

**Keywords:** *Aspergillus terreus* YPGA10, deep-sea-derived fungus, butenolide derivatives, *α*-glucosidase

## Abstract

Three new butenolide derivatives, namely aspernolides N–P (**1**–**3**), together with six known analogues (**4**–**9**), were isolated from the ethyl acetate (EtOAc) extract of the deep sea-derived fungus *Aspergillus terreus* YPGA10. The structures of compounds **1**–**3** were determined on the basis of comprehensive analyses of the nuclear magnetic resonance (NMR) and mass spectroscopy (MS) data, and the absolute configurations of **1** and **2** were determined by comparisons of experimental electronic circular dichroism (ECD) with calculated ECD spectra. Compound **1** represents the rare example of *Aspergillus*-derived butenolide derivatives featured by a monosubstituted benzene ring. Compounds **6**–**9** exhibited remarkable inhibitory effects against *α*-glucosidase with IC_50_ values of 3.87, 1.37, 6.98, and 8.06 μM, respectively, being much more active than the positive control acarbose (190.2 μM).

## 1. Introduction

**Figure 1:**
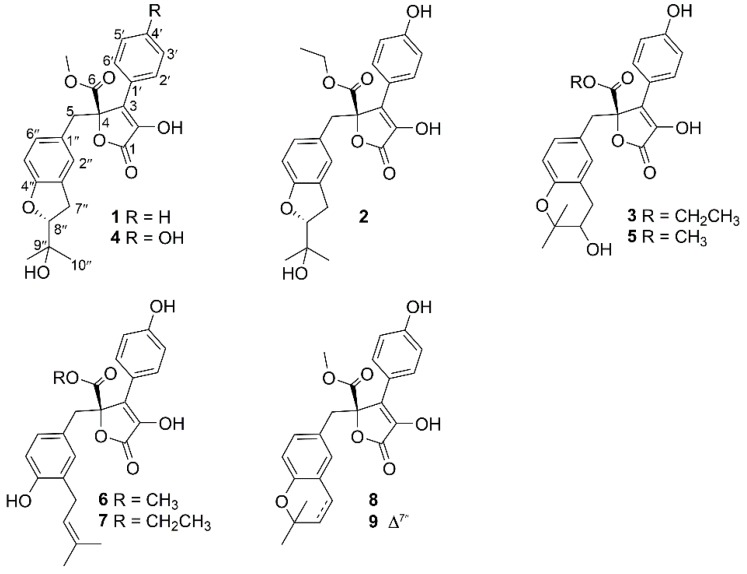
Structures of compounds **1**–**9** from *Aspergillus terreus* YPGA10.

Butyrolactone derivatives from the fungal genera *Aspergillus* are a group of natural products usually consisting of three moieties: An α,β-unsaturated-γ-lactone moiety and two phenyl moieties. These are mainly produced by the species *Aspergillus terreus.* Since butyrolactone I was first reported in 1907, dozens of analogues have been isolated and characterized from the fungal genera *Aspergillus* [1,2,3,4,5,6,7,8,9,10,11]. Some members exhibited significant bioactivity, such as anti-neuroinflammatory activity [5,8], *α*-glucosidase inhibitory activity [9,10], antiplasmodial activity [7], and antibacterial activity [7]. In recent years, deep-sea fungi have been well recognized as a rich source of secondary metabolites endowed with unusual structures and significant bioactivities [12]. As part of our ongoing efforts to discover bioactive molecules from deep-sea derived fungi [13,14,15,16,17], an EtOAc extract of a fungal strain *Aspergillus terreus* YPGA10 displayed the ^1^H NMR resonances similar to those of butyrolactone I. A bioassay revealed that the EtOAc extract possessed an inhibition rate of 67% at a single dose (100 μg/mL) against *α*-glucosidase. Subsequent chromatography of the EtOAc fraction yielded three new butenolide derivatives, namely aspernolides N–P (**1**–**3**), along with six known analogues (**4**–**9**) (Figure 1). All compounds were tested for their inhibitory activities against *α*-glucosidase. Herein, the details of the isolation, structural elucidation, and the *α*-glucosidase inhibitory activities of **1**–**9** are described.

## 2. Results

**Figure 2:**
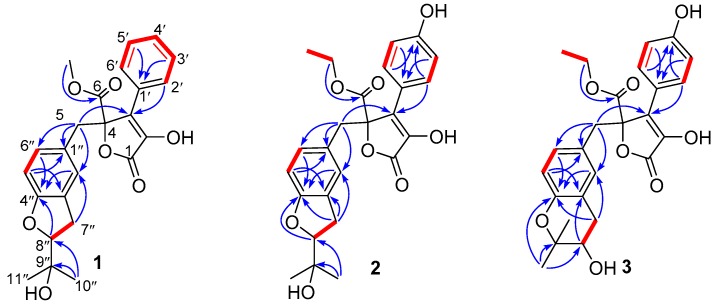
Key correlation spectroscopy (COSY) and heteronuclear multiple bond correlation (HMBC) of **1**–**3**.

**Figure 3:**
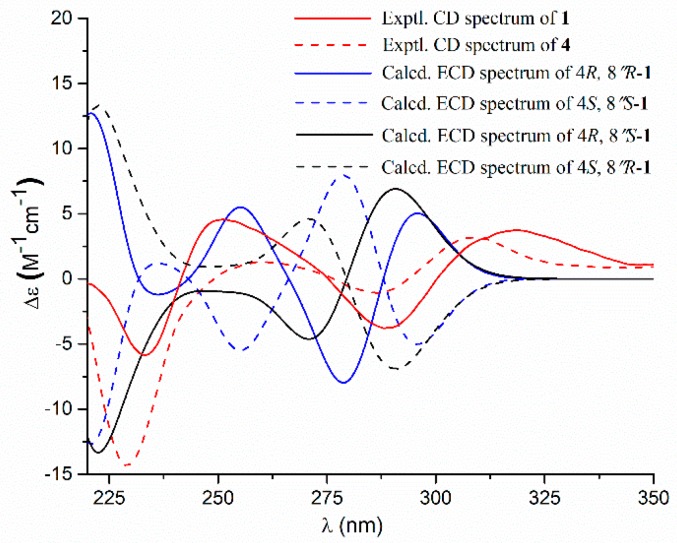
Experimental and calculated electronic circular dichroism (ECD) spectra of **1** and experimental ECD spectrum of **4** in methanol.

Compound **1** had a molecular formula of C_24_H_24_O_7_, as established by the high-resolution electrospray ionization mass spectroscopy (HRESIMS) and nuclear magnetic resonance (NMR) data (Table 1), requiring thirteen degrees of unsaturation. The ^1^H NMR spectrum (Figure S1) provided signals for two methyls (δ_H_ 1.19, s; 1.16, s), a methoxyl (δ_H_ 3.79, s), an oxygenated methine [δ_H_ 4.50 (dd, *J* = 9.3, 8.6 Hz, 1H)], a 1,3,4-trisubstituted benzene ring [δ_H_ 6.59 (d, *J* = 1.6 Hz, 1H); 6.45 (d, *J* = 8.2 Hz, 1H); 6.52 (dd, *J* = 8.2, 1.6 Hz, 1H)], a monosubstituted benzene ring [δ_H_ 7.70 (dd, *J* = 8.4, 1.2 Hz, 2H); 7.46 (dd, *J* = 8.4, 7.4 Hz, 2H); 7.37 (dd, *J* = 7.4, 1.2 Hz, 1H)], and two methylenes (δ_H_ 2.99, m; 3.49, s). While the ^13^C NMR and the heteronuclear single quantum coherence (HSQC) spectra (Figures S2 and S3) exhibited 24 carbon resonances attributable to two benzene rings (δ_C_ 132.1, 128.5, 128.5, 129.8, 129.8, 129.6, 126.2, 127.9, 128.1, 160.5, 109.1, 131.0), a double bond [δ_C_ 128.1, C-2 (not detected)], two carbonyls (δ_C_ 170.3, 171.5), two methylenes (δ_C_ 39.6, 31.3), a methoxy group (δ_C_ 53.9), an oxygenated methine (δ_C_ 90.4), and a oxygenated tertiary carbon (δ_C_ 72.5). The ^1^H and ^13^C NMR data in association with the heteronuclear multiple bond correlation (HMBC) correlations established a butenolide derivative, structurally related to a co-isolated known compound butyrolactone IV (**4**) [6]. The only difference was owing to the presence of a monosubstituted benzene ring in **1** instead of the 1,4-disubstituted benzene ring in **4**. The structure of **1** was further secured by detailed analyses of the 2D NMR data (Figure 2). In order to assign the absolute configuration, the ECD calculation was performed at the b3lyp/6-31+g(d,p) level in methanol using the b3lyp/6-31+g(d,p)-optimized geometries for the four possible model molecules. The theoretical ECD spectra for (4*R*, 8″*R*)-**1**, (4*R*, 8″*S*)-**1**, and their enantiomers were calculated by the time-dependent density functional theory (TDDFT) method. Comparison of the experimental CD curve of **1** with the computed ECD curves (Figure 3) indicated the absolute configurations of **1** to be 4*R* and 8″*R*. The absolute configuration of **1** was supported by possessing similar specific rotation and CD spectrum to those of **4**, whose structure was established by X-ray single-crystal diffraction experiment [6]. Compound **1**, featured by a monosubstituted benzene ring, was rarely found in this class of butenolide derivatives and was named aspernolide N.

**Figure 4:**
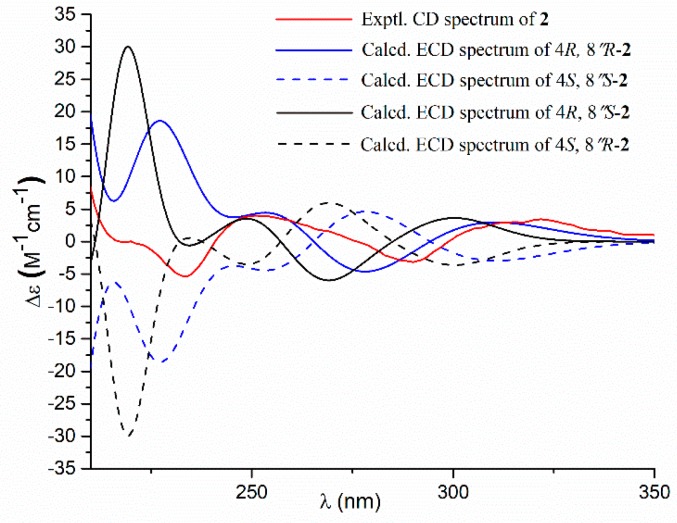
Experimental and calculated electronic circular dichroism (ECD) spectra of **2** in methanol.

Compound **2** had a molecular formula of C_25_H_26_O_8_, as established by the HRESIMS data (Figure S28), requiring 13 degrees of unsaturation. The ^1^H NMR and ^13^C NMR data (Table 1) were almost identical with those of **4**, with the only distinction attributable to the presence of an ethoxy group [δ_H_ 4.25 (2H, q, *J* = 7.1 Hz), 1.21 (3H, t, *J* = 7.1 Hz); δ_C_ 63.7] in **2** instead of the methoxy group [δ_H_ 3.76 (3H, s); δ_C_ 53.9] in **4**. The linkage of the ethoxy group to C-6 was deduced by the COSY relationship between the methyl protons (δ_H_ 1.21) and the oxygenated methyene protons (δ_H_ 4.25) in combination with the HMBC correlations from the methyene protons (δ_H_ 4.25) to the carbonyl carbon C-6 (δ_C_ 171.0) (Figure 2). Comparison of the experimental ECD spectra with the calculated ECD data for the model molecules (4*R*,8″*R*; 4*S*,8″*S*; 4*R*, 8″*S*; 4*S*, 8″*R*) allowed the assignment of the 4*R* and 8″*R* configurations for **2** (Figure 4).

**Figure 5:**
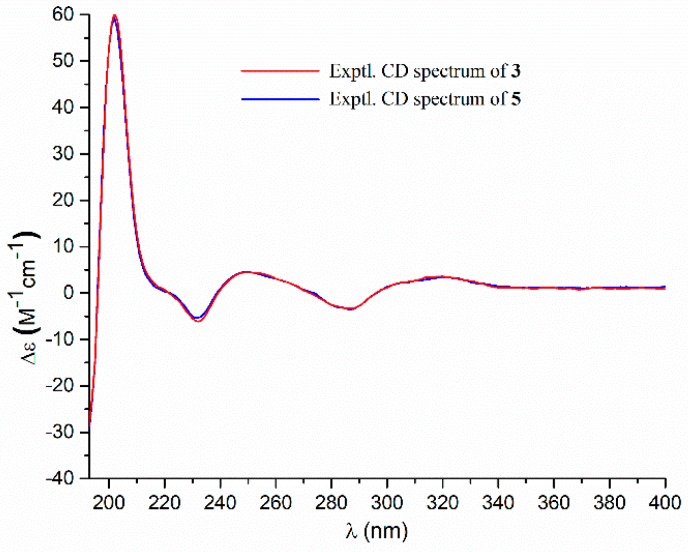
Experiment ECD spectra of **3** and **5** in methanol.

Compound **3** had a molecular formula of C_25_H_26_O_8_, as established by the HRESIMS data (Figure S29), requiring 13 degrees of unsaturation. The ^1^H NMR and ^13^C NMR data (Table 1) provided the characteristic resonances for a 1,3,4-trisubstituted benzene ring, a 1,4-disubstituted benzene ring, and an α,β-unsaturated-γ-lactone group. Analyses of the 2D NMR spectra conducted **3** to be an analogue of a co-isolated known compound butyrolactone V (**5**) [18]. The difference was found by the presence of an ethoxy group [δ_H_ 4.25 (2H, q, *J* = 7.0 Hz), 1.21 (3H, t, *J* = 7.0 Hz); δ_C_ 63.7] in **3** instead of the methoxy group [δ_H_ 3.78 (3H, s); δ_C_ 53.9] in **5**. The ethoxy group was located at C-6 by the HMBC correlations from the oxygenated methyene protons (δ_H_ 4.25) to the carbonyl carbon C-6 (δ_C_ 171.0). The structure of **3** was further secured by detailed analyses of 2D NMR data (Figure 2). The abosulte configuration of C-4 was proposed to be *R*, the same as that of **5,** based on their almost identical CD spectra (Figure 5). Compound **3** was given the trivial name aspernolide P.

In addition, six additional known compounds were identical to butyrolactone IV (**4**) [6], butyrolactone V (**5**) [18], butyrolactone I (**6**) [3], butyrolactone VII (**7**) [7], aspernolide A (**8**) [1], and aspernolide E (**9**) [2] based on comparisons of their NMR data (Figures S16–S26) and specific rotations with those reported in the literature.

As the extract exhibited strong inhibitions against *α*-glucosidase, and the literature suggested that some members of this class of butyrolactone derivatives possessed significant inhibitions against *α*-glucosidase [9,10]. Thus, all compounds were screened for their inhibitory activities against *α*-glucosidase (Table 2) at the initial concentration of 100 μM. Compounds **6**–**9** with inhibitions more than 50% were further evaluated to calculate the IC_50_ values (Table 2). The results showed that compounds **6**–**9** were strong inhibitors with IC_50_ values ranging from 1.37 to 8.06 μM, being more active than the positive control acarbose (190.2 μM). The structural variability of this series of butyrolactone derivatives and their inhibitory activity toward *α*-glucosidase in our study may define some structure-activity relationship: (a) The ethoxyl group at C-6 may lead to a small increase of the activity than methoxyl group, as compound **7** was 2-fold more active than compound **6**. (b) The Δ^7”^ of the pyran ring may have negligible effects on the activity, as compound **9** possessed similar activity to its hydrogenated derivative **8**. (c) The introduction of a hydroxy group at C-8” of the pyran ring may lead a sharp decrease in activity, as compound **8** exhibited significant inhibition at 100 μM, while its 8”-hydroxylated derivative **5** showed negligible inhibitory activity at the same concentration.

The *α*-glucosidase inhibitory activities of **1**–**3**, **7**, and **9** were evaluated for the first time, and the preliminary structure−activity relationship may provide information for further structural optimization of these *α*-glucosidase inhibitors.

## 3. Experimental Section

### 3.1. General Experimental Procedure

Specific rotations were measured by an SGW^®^-1 automatic polarimeter (Shanghai Jing Ke Industrial Co., Ltd., Shanghai, China). Ultraviolet (UV) spectra were measured on a UV-2600 spectrometer. ECD spectra were measured on an Aviv Model 420SF spectropolarimeter (Aviv Biomedical Inc., Lakewood, CA, USA). The NMR spectra were recorded on a Bruker Avance III HD-400 spectrometer (Bruker, Fällanden, Switzerland). HRESIMS spectra were obtained on a Waters Xevo G2 Q-TOF spectrometer (Waters Corporation, Milford, MA, USA). Semi-preparative high-performance liquid chromatography (HPLC) was undertaken on a Shimadzu LC-6AD pump (Shimadzu Co., Kyoto, Japan) using a UV detector, and a YMC-Pack ODS-A HPLC column (semipreparative, 250 × 10 mm, S-5 μM, 12 nm, YMC Co., Ltd., Kyoto, Japan) was used for separation.

### 3.2. Fungal Strain and Identification

Fungus *Aspergillus terreus* YPGA10 was isolated from the deep-sea water at a depth of 4159 m in the Yap Trench (West Pacific Ocean). The strain was identified as *Aspergillus terreus* based on microscopic examination and by internal transcribed spacer (ITS) sequencing. The ITS sequence has been deposited in GenBank (http://www.ncbi.nlm.nih.gov) with accession number MG835907. The strain YPGA10 (MCCC3A01013) was deposited at the Marine Culture Collection of China.

### 3.3. Fermentation

The fermentation was carried out in 40 Fernbach flasks (500 mL), each containing 80 g of rice. Distilled water (90 mL) was added to each flask, and the contents were soaked for 3 h before autoclaving at 15 psi for 30 min. After cooling to room temperature, each flask was inoculated with 3.0 mL of the spore inoculum and incubated at room temperature for 30 days.

### 3.4. Extraction and Isolation

The fermented materials were extracted with EtOAc (3 × 2000 mL) in an ultrasonic bath at 25 °C for 30 min. After evaporation under vacuum, the EtOAc extract (12.0 g) was subjected to ODS silica gel column chromatography (CC) eluting with MeOH/H_2_O (20:80→100:0) to afford 10 fractions (F1–F10). F7 was further chromatographed over C-18 silica gel CC eluted with MeOH/H_2_O (65:35) to afford 7 subfractions (F7a–F7g). F7d was further purified by HPLC on a semi-preparative YMC-pack ODS-A column using MeOH/H_2_O (65:35, 2 mL/min) to afford **7** (71 mg, t*_R_* 45 min). F7e was separated by HPLC using MeOH/H_2_O (67:33, 2 mL/min) to give **1** (1.4 mg, t*_R_* 44 min). F7f was separated by HPLC using ACN/H_2_O (57:43, 2 mL/min) to obtain **8** (31 mg, t*_R_* 65 min) and **9** (1.4 mg, t*_R_* 57 min). F6 was subjected to sephadex LH-20 (MeOH) to obtain F6a–F6e and **6** (1.8 g). F6b was further chromatographed over C-18 silica gel CC eluted with MeOH/H_2_O (40%, 50%, 60%, 70%, 80%, 90%, 100%) to give F6b1–F6b5. F6b1 was separated by HPLC eluted with ACN/H_2_O (50:50, 2 mL/min) to afford **4** (101 mg, t*_R_* 43 min). F6b2 was chromatographed by HPLC using ACN/H_2_O (51:49, 2 mL/min) as eluent to obtain **3** (7 mg, t*_R_* 44 min) and **2** (10 mg, t*_R_* 46 min). F6b3 was purified by HPLC eluted with ACN/H_2_O (47:53, 2 mL/min) to afford **5** (23 mg, t*_R_* 38 min).

Aspernolide N (**1**): Colorless oil; [α]${}_{D}^{25}$ +59 (*c* 0.03, MeOH); UV (MeOH) λ_max_ (log *ε*) 201 (4.39), 287 (3.73) nm; ECD (*c* 2.8 × 10^−4^ M, MeOH) λ_max_ (Δε) 286 (−1.09), 229 (−14.39), 202 (+43.55); ^1^H and ^13^C NMR data, see Table 1; HRESIMS *m*/*z* 447.1403 [M + Na]^+^ (calcd. for C_24_H_24_O_7_Na^+^, 447.1414).
Aspernolide O (**2**): Colorless oil; [α]${}_{D}^{25}$ +63 (*c* 0.16, MeOH); UV (MeOH) *λ*_max_ (log *ε*) 226 (4.15), 306 (4.33) nm; ECD (*c* 2.2 × 10^−4^ M, MeOH) λ_max_ (Δε) 290 (−3.16), 251 (+3.99), 235 (−5.35), 202 (+47.49); ^1^H and ^13^C NMR data, see Table 1; HRESIMS *m*/*z* 477.1525 [M + Na]^+^ (calcd. for C_25_H_26_O_8_Na^+^, 477.1520).
Aspernolide P (**3**): Colorless oil; [α]${}_{D}^{25}$ +73 (*c* 0.14, MeOH); UV (MeOH) *λ*_max_ (log *ε*) 226 (4.33), 306 (4.43) nm; ECD (*c* 2.1 × 10^−4^ M, MeOH) λ_max_ (Δε) 286 (−3.32), 249 (+4.45), 232 (−6.16), 202 (+60.34); ^1^H and ^13^C NMR data, see Table 1; HRESIMS *m*/*z* 477.1518 [M + Na]^+^ (calcd. for C_25_H_26_O_8_Na^+^, 477.1520).
Butyrolactone IV (**4**): ECD (*c* 2.2 × 10^−4^ M, MeOH) λ_max_ (Δε) 288 (−3.78), 233 (−5.86), 203 (+48.20).
Butyrolactone V (**5**): ECD (*c* 2.8 × 10^−4^ M, MeOH) λ_max_ (Δε) 286 (−3.50), 249 (+4.61), 232 (−5.31), 202 (+59.41).

### 3.5. Computation Section

In general, conformational analyses were carried out via random searching in the Sybyl-X 2.0 [19] using the MMFF94S force field with an energy cutoff of 2.5 kcal/mol. Subsequently, the conformers were re-optimized using density functional theory (DFT) at the b3lyp/6-31+g(d,p) level in MeOH using the polarizable conductor calculation model by the GAUSSIAN 09 program [20]. The energies, oscillator strengths, and rotational strengths (velocity) of the first 30 electronic excitations were calculated using the TDDFT methodology at the rcam-b3lyp/6-31+g(d,p) level in MeOH. The ECD spectra were simulated by the overlapping Gaussian function (half the bandwidth at 1/e peak height, σ = 0.16 for (4*R*, 8″*R*) and (4*R*, 8″*S*)-**1**, 0.30 for (4*R*, 8″*R*)-**2**, and 0.2 for (4*R*, 8″*S*)-**2**). By comparing the experiment spectra with the calculated ECD spectra, the absolute configurations of **1** and **2** were resolved.

### 3.6. α-Glucosidase Assay

The *α*-glucosidase inhibitory effect was assessed using a previously described method with slight modification [21,22]. 0.2 U of *α*-glucosidase from *Saccharomyes cerevisiae* purchased from Sigma-Aldrich (St. Louis, MO, USA) was diluted to 0.1 M phosphate buffer consisting of Na_2_HPO_4_ and NaH_2_PO_4_ (pH 6.8). The assay was conducted in a 200 μL reaction system containing 148 μL of the buffer, 25 μL of diluted enzyme solution, and 2 μL of DMSO or sample (dissolved in DMSO). After 20 min of incubation in the 96-well plates at 37 °C, 25 μL of 0.4 mM 4-nitrophenyl-β-d-glucopyranoside (PNPG, Aladdin, Shanghai, China) was added as a substrate to start the enzymatic reaction. The plate was incubated for an additional 15 min at 37 °C, followed by the measurement of the optical density (OD). The final concentrations of tested compounds were between 0.39 and 100 μM. The OD was measured at an absorbance wavelength of 405 nm using a Microplate Reader (Tecan, Switzerland). All assays were performed in three replicates, and acarbose (Aladdin, Shanghai, China) was used as the positive control.

## 4. Conclusions

In conclusion, three new butenolide derivatives (**1**–**3**) and six known analogues (**4**–**9**) were isolated from the EtOAc extract of the strain *Aspergillus terreus* YPGA10, a fungus isolated from deep-sea sediments. The structures of compounds **1**–**3** were determined on the basis of comprehensive analyses of the NMR and MS data, and the absolute configurations of **1** and **2** were determined by comparisons of experimental ECD with calculated ECD spectra. Compound **1**, possessing a monosubstituted benzene ring, is rarely found in this group of butenolide derivatives. Compounds **6**–**9** exhibited remarkable inhibitory effects against *α*-glucosidase with IC_50_ values of 3.87, 1.37, 6.98, 8.06 μM, respectively, being much more active than the positive control acarbose (190.2 μM), which suggested that they could be developed as potential inhibitors of *α*-glucosidase.

## Figures and Tables

**Table 1:** ^1^H and ^13^C NMR Data of **1**–**3** in Methanol-*d*_4_^a^.

No.	1		2		3	
	δ_H_	δ_C_	δ_H_	δ_C_	δ_H_	δ_C_
1		170.3		170.5		170.7
2		not detected		140.0		140.2
3		128.1		129.1		129.0
4		86.9		86.9		86.9
5	3.49, s	39.6	3.46, s	39.7	3.44, s	39.5
6		171.5		171.0		171.0
1′		132.1		123.2		123.3
2′, 6′	7.70, dd (8.4, 1.2)	128.5	7.60, d (8.9)	130.4	7.59, d (8.7)	130.3
3′, 5′	7.46, dd (8.4, 7.4)	129.8	6.87, d (8.9)	116.6	6.87, d (8.7)	116.5
4′	7.37, dd (7.4, 1.2)	129.6		159.3		159.2
1″		126.2		126.3		126.2
2″	6.59, d (1.6)	127.9	6.61, d (1.5)	127.9	6.48, d (1.8)	132.9
3″		128.1		128.0		120.5
4″		160.5		160.4		153.4
5″	6.45, d (8.2)	109.1	6.46, d (8.2)	109.1	6.48, d (8.2)	117.2
6″	6.52, dd (8.2, 1.6)	131.0	6.54, dd (8.2, 1.5)	131.0	6.56, dd (8.5, 1.8)	130.4
7″	2.99, m	31.3	2.99, m	31.3	2.77, dd (16.6, 5.4); 2.53, dd (16.6, 7.5)	32.0
8″	4.50, dd (9.3, 8.6)	90.4	4.49, dd (9.3, 8.6)	90.4	3.67, dd (7.5, 5.4)	70.4
9″		72.5		72.5		77.9
10″	1.19, s	25.2	1.19, s	25.2	1.27, s	25.8
11″	1.16, s	25.3	1.16, s	25.3	1.17, s	20.9
OCH_2/3_	3.79, s	53.9	4.25, q (7.1)	63.7	4.25, q (7.0)	63.7
CH_3_			1.21, t (7.1)	14.2	1.21, t (7.0)	14.2

*^a^*^1^H NMR recorded at 400 MHz, ^13^C NMR recorded at 100 MHz.

**Table 2:** Inhibitory Effects of the Compounds on *α*-Glucosidase.

No.	%Inhibition (100 μM)	IC_50_ (μM)
**1**	18.62	- ^c^
**2**	23.18	- ^c^
**3**	26.43	- ^c^
**4**	37.29	- ^c^
**5**	21.57	- ^c^
**6**	100/89.41 ^b^	3.87 ± 0.33
**7**	100/98.69 ^b^	1.37 ± 0.05
**8**	89.17	6.98 ± 0.22
**9**	90.43	8.06 ± 0.21
Acarbose ^a^		190.2 ± 2.4

^a^ Positive control. ^b^ 50 μM. ^c^ Not tested.

## Supplementary Materials

The following are available online at https://www.mdpi.com/1660-3397/17/6/332/s1, Figures S1–S29: ^1^H, ^13^C NMR, HSQC, ^1^H-^1^H COSY, HMBC, HRESIMS spectra of the new compounds **1**–**3**, ^1^H and ^13^C NMR of known compounds **4**–**9**.
