# Supplementary material for: Butenolide Derivatives with α-Glucosidase Inhibitions from the Deep-Sea-Derived Fungus Aspergillus terreus YPGA10
Source: Mar Drugs. 2019 Jun 3;17(6):332. doi: 10.3390/md17060332 (PMC6627487; doi:10.3390/md17060332)
Supplement: Supplementary file 1 [file marinedrugs-17-00332-s001.pdf]

Butenolide Derivatives with α-Glucosidase Inhibitions from the Deep-Sea-Derived Fungus *Aspergillus terreus* YPGA10

Zhongbin Cheng [1,†], Yuanli Li [1,†], Wan Liu [1], Lijun Liu [1], Jie Liu [1], Wangjun Yuan [1,*], Zhuhua Luo [2], Wei Xu [2,*] and Qin Li [1,3,*]

[1] Pharmaceutical College, Henan University, Kaifeng 475004, China; czb360@126.com (Z.C.); lyl3287439993@163.com (Y.L.); 18737806806@163.com (W.L.); 15736871748@163.com (L.L.); ll18737801136@163.com (J.L.)
[2] Key Laboratory of Marine Biogenetic Resources, Third Institute of Oceanography, Ministry of Natural Resources, Xiamen 361005, China
[3] Eucommia Ulmoides Cultivation and Utilization of Henan Engineering Laboratory, Kaifeng 475004, China
* Correspondence: yuanwangjun@henu.edu.cn (W.Y.); xuwei@tio.org.cn (W.X.); liqin6006@163.com (Q.L.); Tel.: +86-371-2388-3849 (Q.L.)
† These authors contributed equally to this work.

	Table of Contents	Page
**Figure S1**	^{1}H NMR Spectrum of **1** in Methanol-d_4 (400 MHz).	1
**Figure S2**	^{13}C NMR Spectrum of **1** in Methanol-d_4 (100 MHz)	1
**Figure S3**	HSQC Spectrum of **1** in Methanol-d_4	2
**Figure S4**	^{1}H-^{1}H COSY Spectrum of **1** in Methanol-d_4	2
**Figure S5**	HMBC Spectrum of **1** in Methanol-d_4	3
**Figure S6**	^{1}H NMR Spectrum of **2** in Methanol-d_4 (400 MHz)	3
**Figure S7**	^{13}C NMR Spectrum of **2** in Methanol-d_4 (100 MHz)	4
**Figure S8**	HSQC Spectrum of **2** in Methanol-d_4	4
**Figure S9**	^{1}H-^{1}H COSY Spectrum of **2** in Methanol-d_4	5
**Figure S10**	HMBC Spectrum of **2** in Methanol-d_4	5
**Figure S11**	^{1}H NMR Spectrum of **3** in Methanol-d_4 (400 MHz)	6
**Figure S12**	^{13}C NMR Spectrum of **3** in Methanol-d_4 (100 MHz)	6
**Figure S13**	HSQC Spectrum of **3** in Methanol-d_4	7
**Figure S14**	^{1}H-^{1}H COSY Spectrum of **3** in Methanol-d_4	7
**Figure S15**	HMBC Spectrum of **3** in Methanol-d_4	8
**Figure S16**	^{1}H NMR Spectrum of **4** in Methanol-d_4 (400 MHz)	8
**Figure S17**	^{13}C NMR Spectrum of **4** in Methanol-d_4 (100 MHz)	9
**Figure S18**	^{1}H NMR Spectrum of **5** in Methanol-d_4 (400 MHz)	9

**Figure S19**	^{13}C NMR Spectrum of **5** in Methanol-d_4 (100 MHz)............	10
**Figure S20**	^{1}H NMR Spectrum of **6** in Methanol-d_4 (400 MHz).............	10
**Figure S21**	^{13}C NMR Spectrum of **6** in Methanol-d_4 (100 MHz)............	11
**Figure S22**	^{1}H NMR Spectrum of **7** in Methanol-d_4 (400 MHz).............	11
**Figure S23**	^{13}C NMR Spectrum of **7** in Methanol-d_4 (100 MHz)............	12
**Figure S24**	^{1}H NMR Spectrum of **8** in Methanol-d_4 (400 MHz).............	12
**Figure S25**	^{13}C NMR Spectrum of **8** in Methanol-d_4 (100 MHz)............	13
**Figure S26**	^{1}H NMR Spectrum of **9** in Methanol-d_4 (400 MHz).............	13
**Figure S27**	HRESIMS spectrum of **1**...	14
**Figure S28**	HRESIMS spectrum of **2**...	14
**Figure S29**	HRESIMS spectrum of **3**...	15

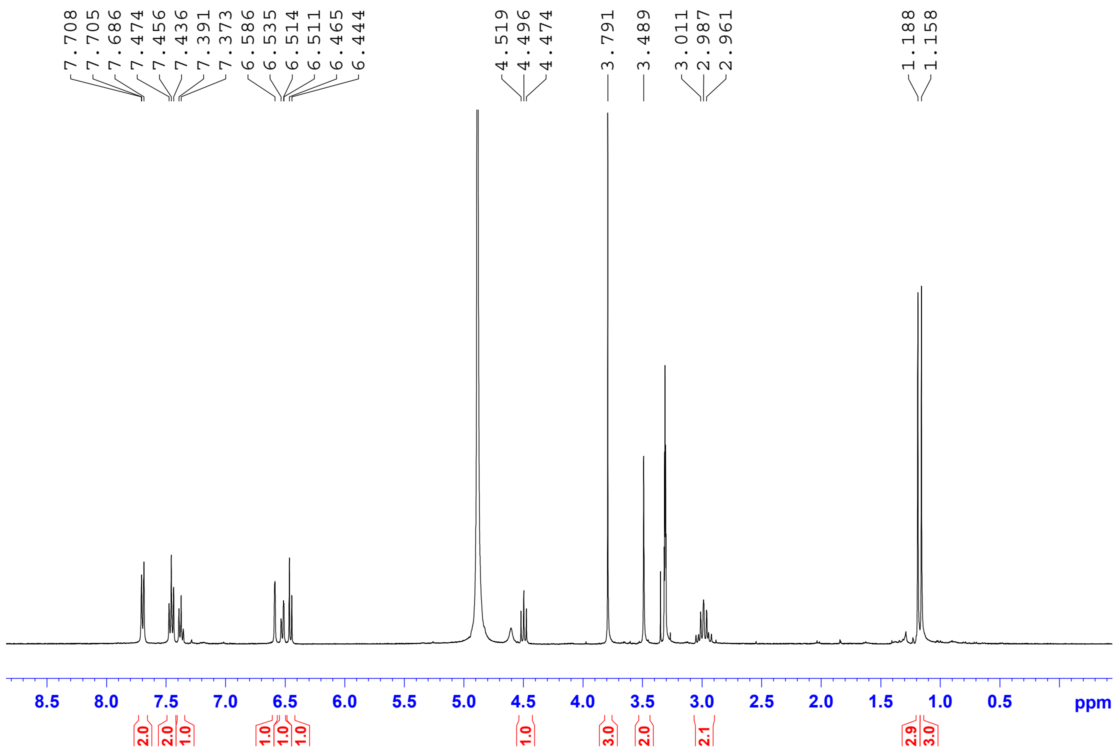

Figure S1 ^{1}H NMR Spectrum of **1** in Methanol-*d*4 (400 MHz)

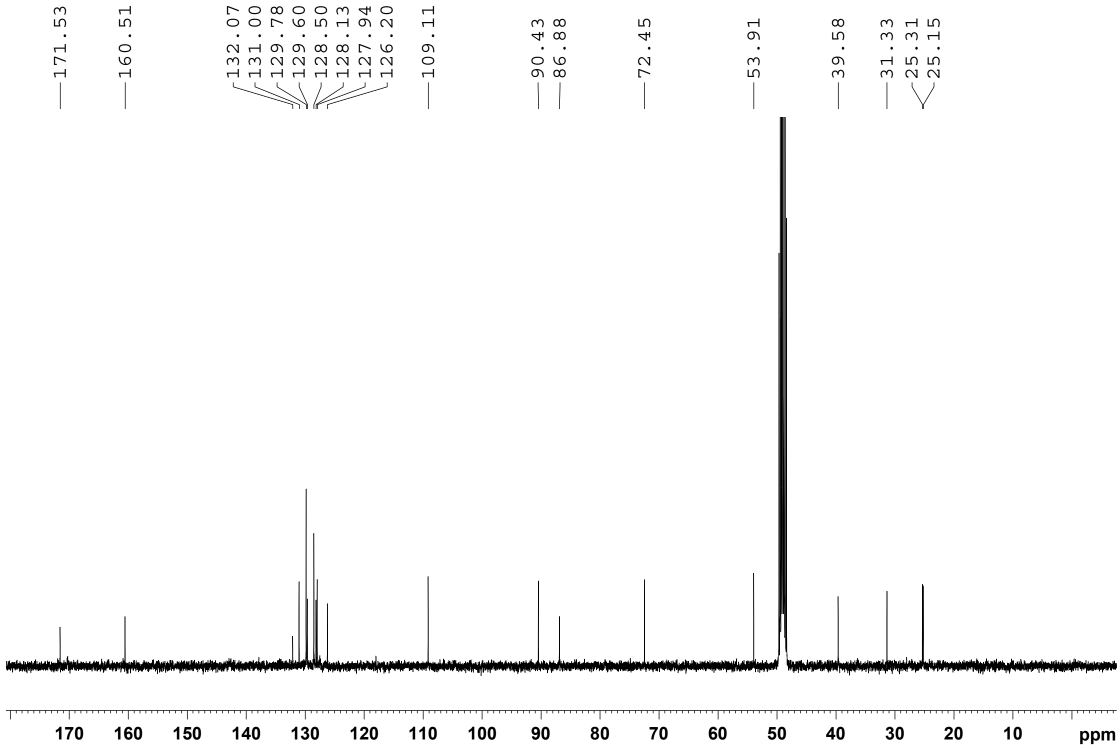

Figure S2 ^{13}C NMR Spectrum of **1** in Methanol-*d*4 (100 MHz)

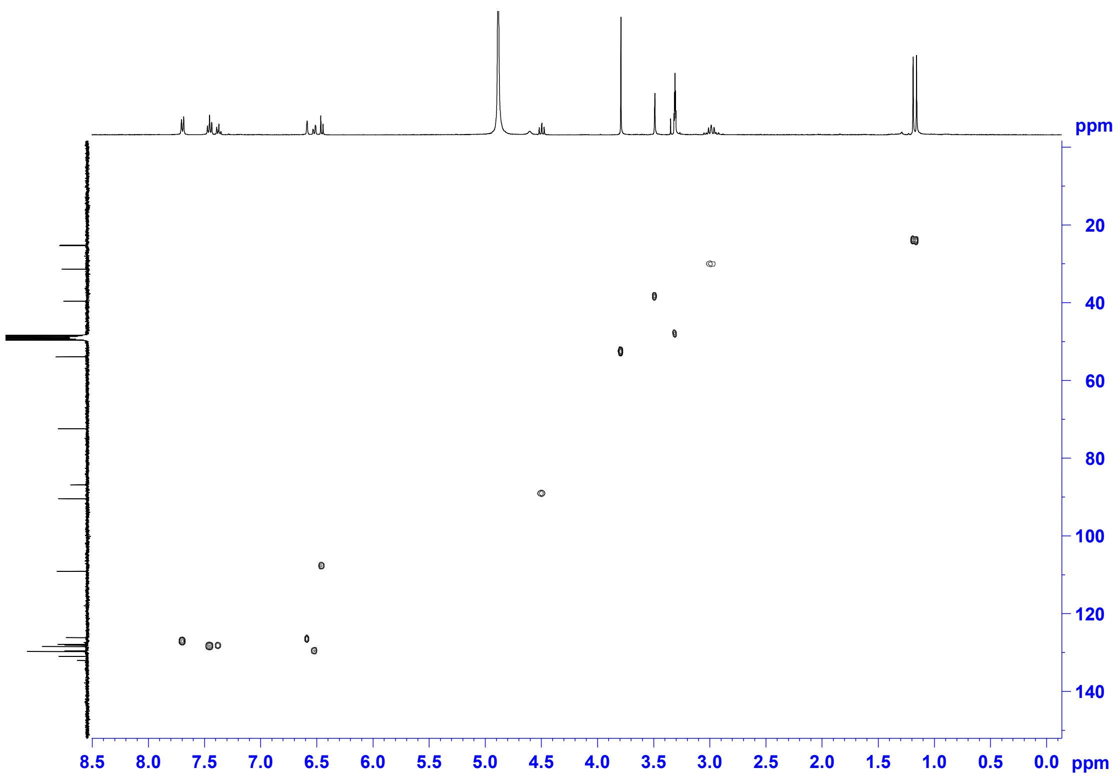

Figure S3 HSQC Spectrum of **1** in Methanol-*d*4

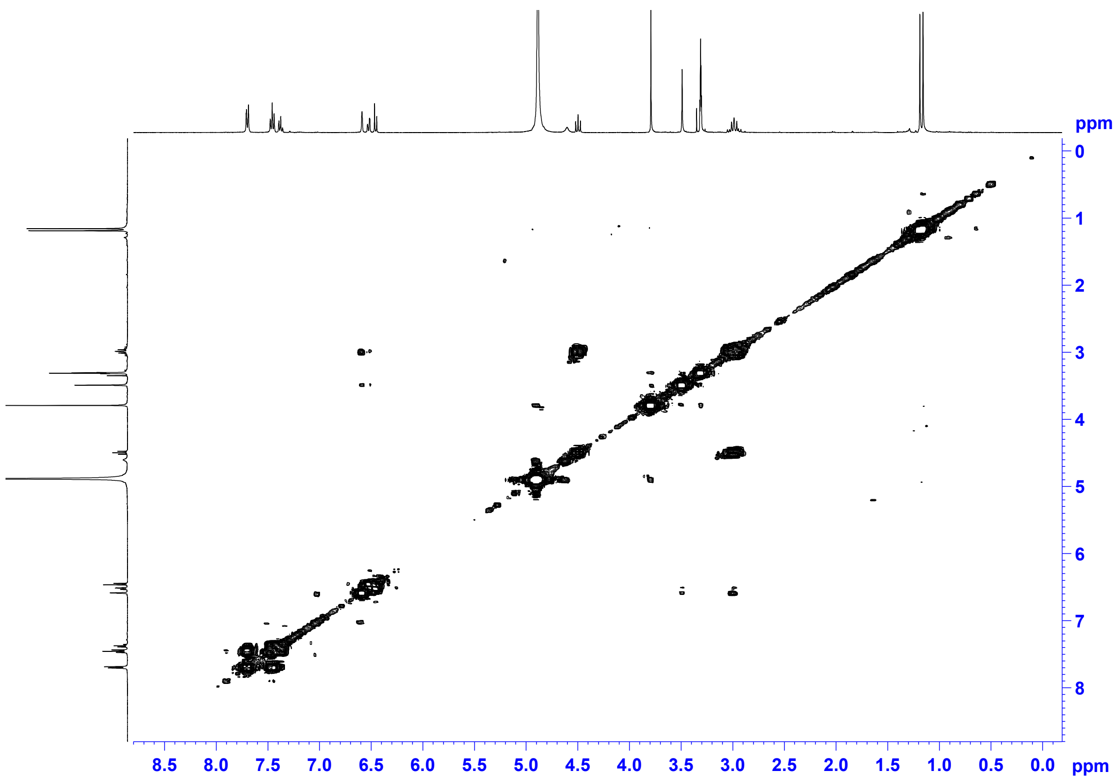

Figure S4 ^{1}H-^{1}H COSY Spectrum of **1** in Methanol-*d*4

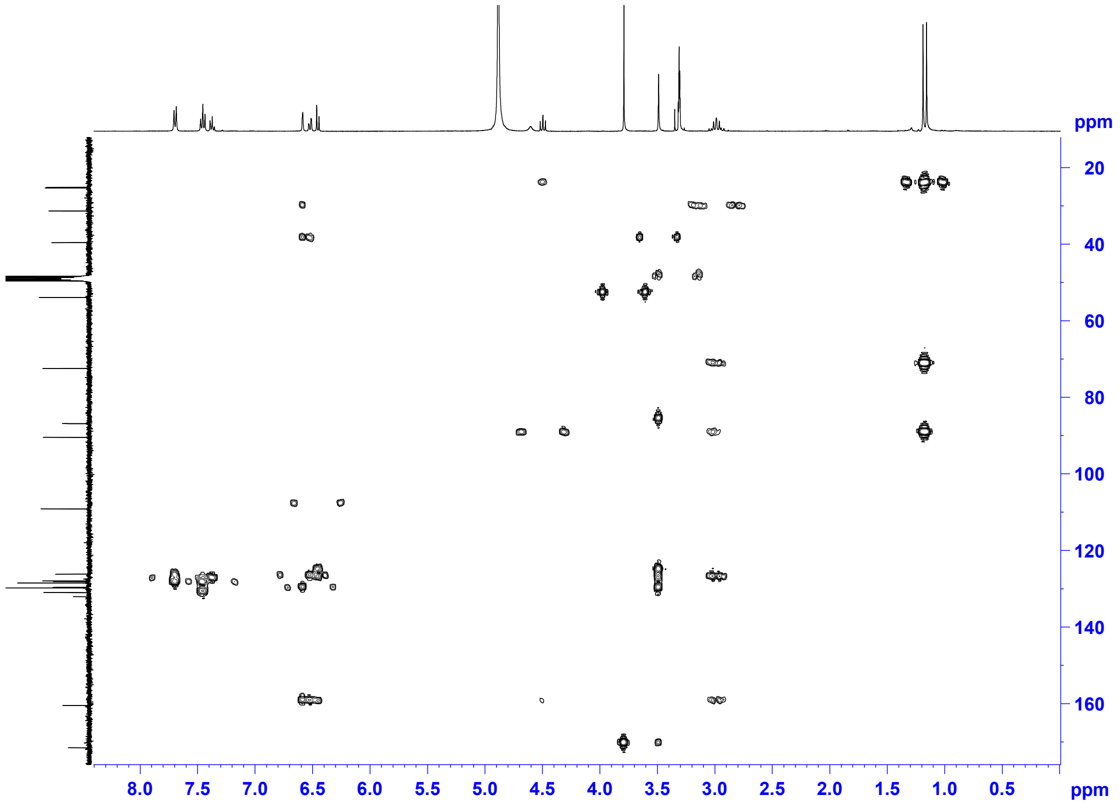

Figure S5 HMBC Spectrum of **1** in Methanol-d_4

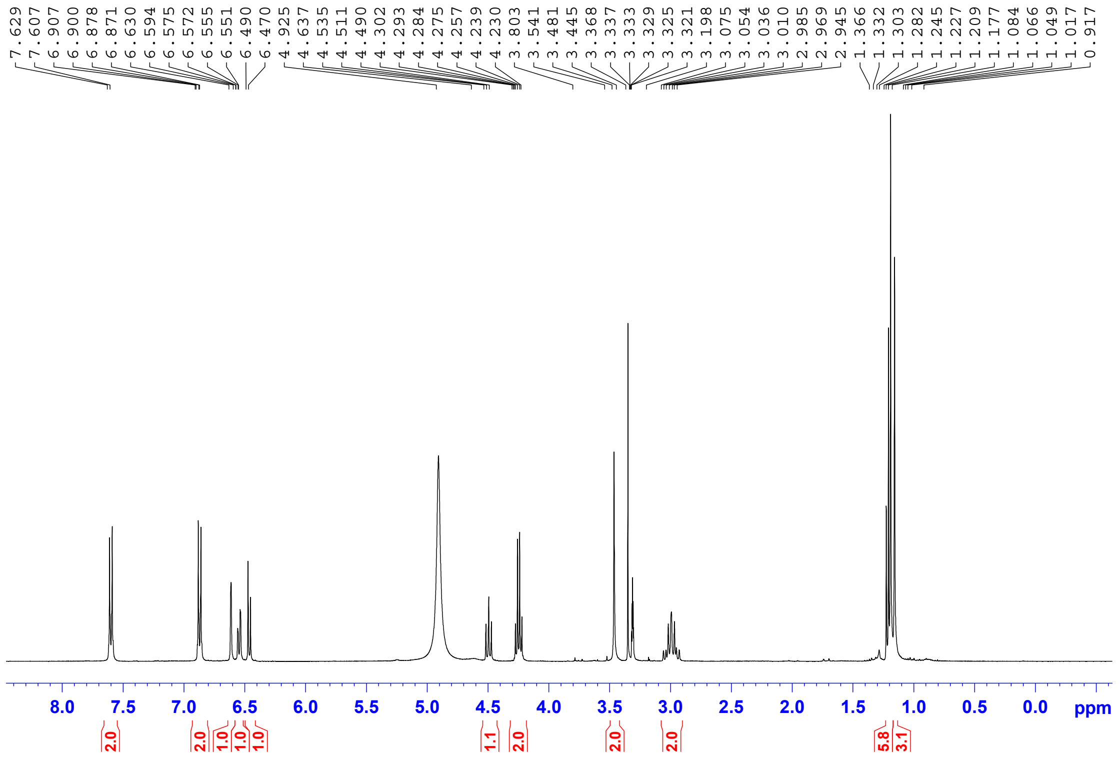

Figure S6 ^{1}H NMR Spectrum of **2** in Methanol-d_4 (400 MHz)

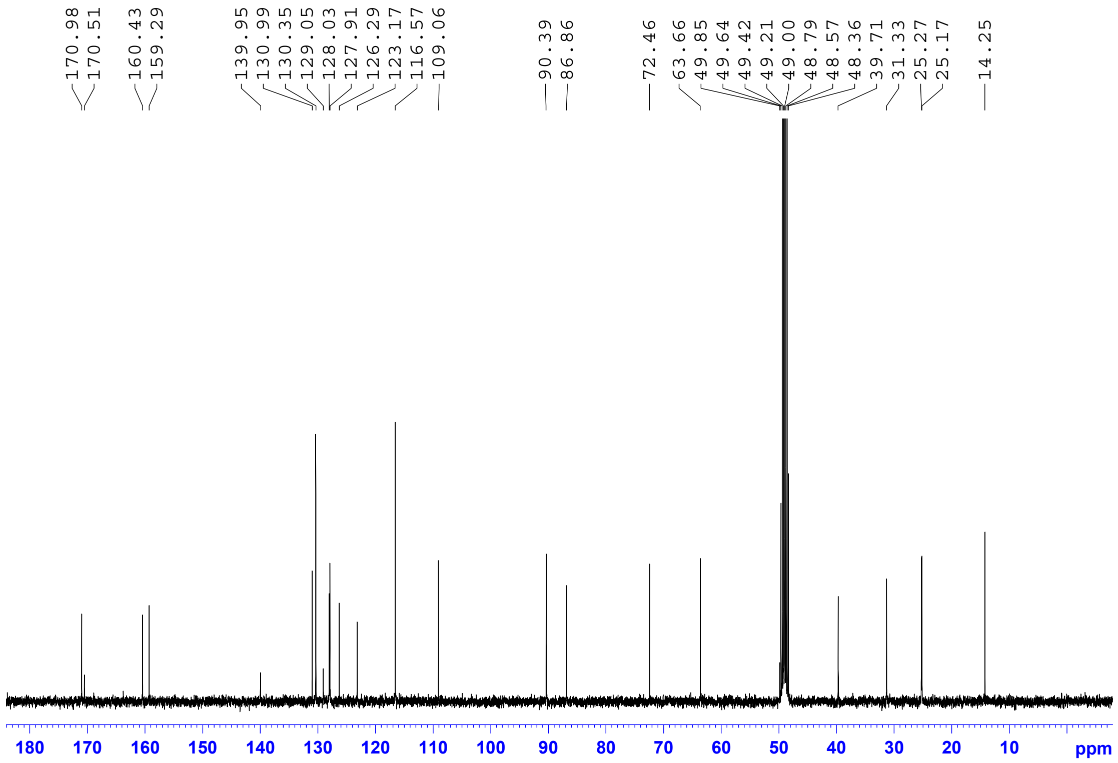

Figure S7 ^{13}C NMR Spectrum of **2** in Methanol-d_4 (100 MHz)

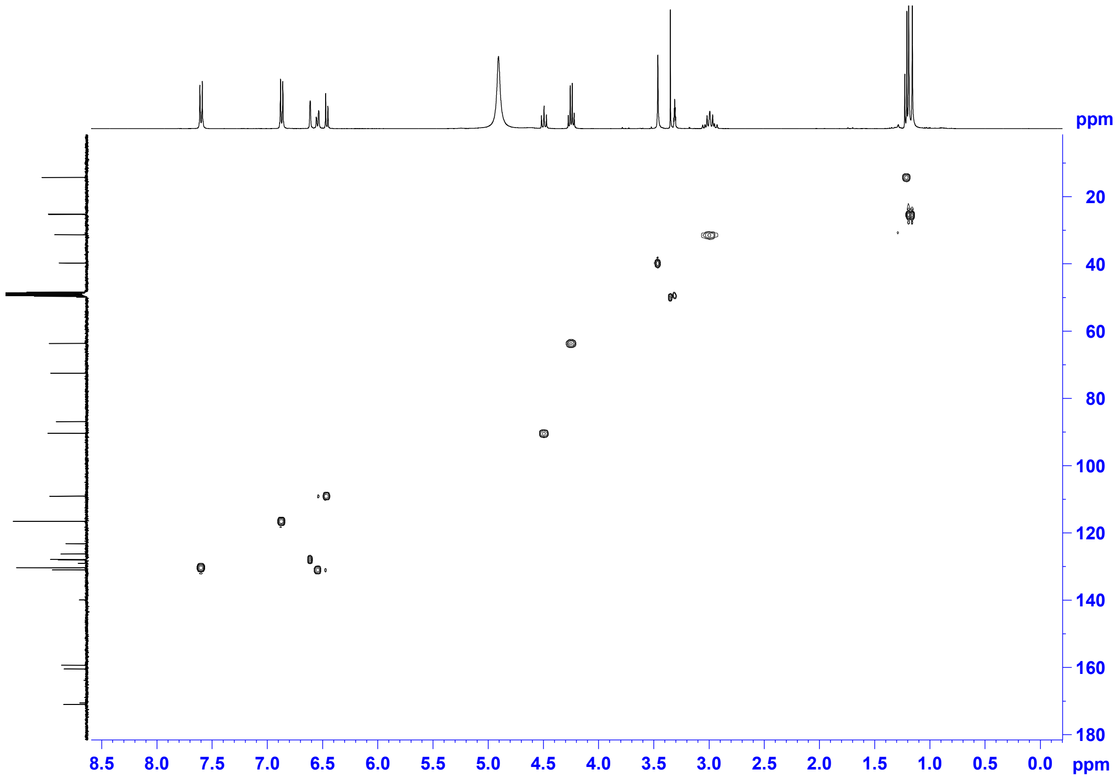

Figure S8 HSQC Spectrum of **2** in Methanol-d_4

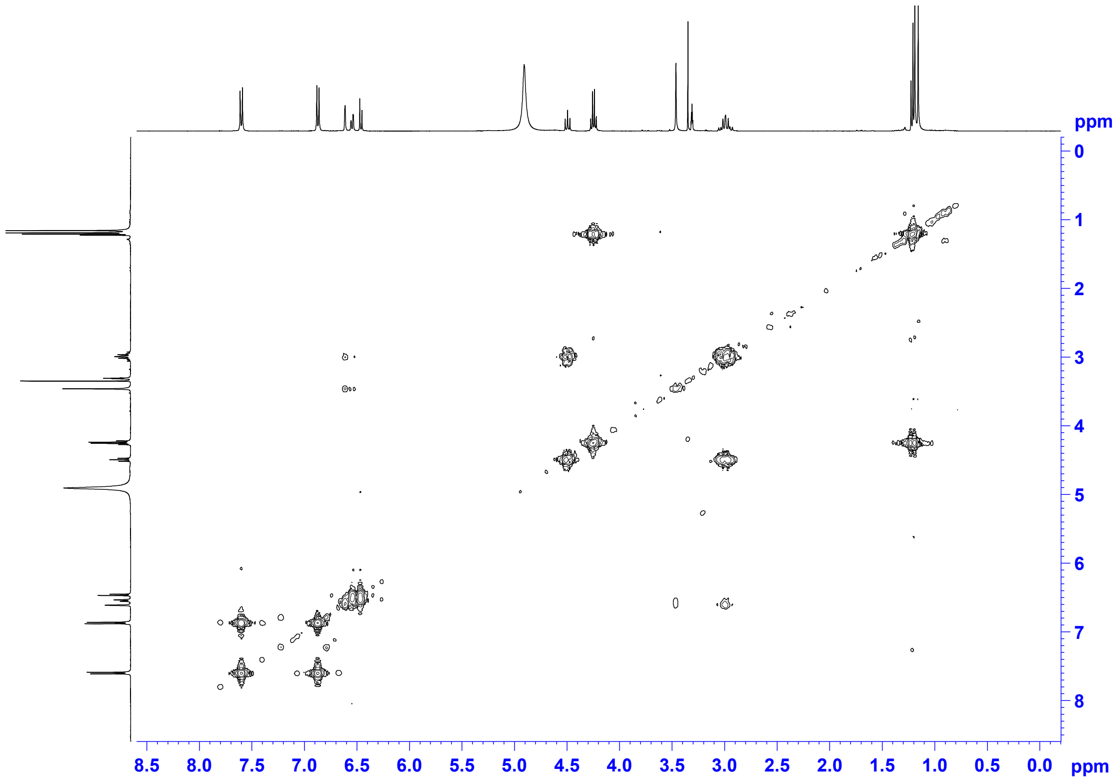

Figure S9 ^{1}H-^{1}H COSY Spectrum of **2** in Methanol-d_4

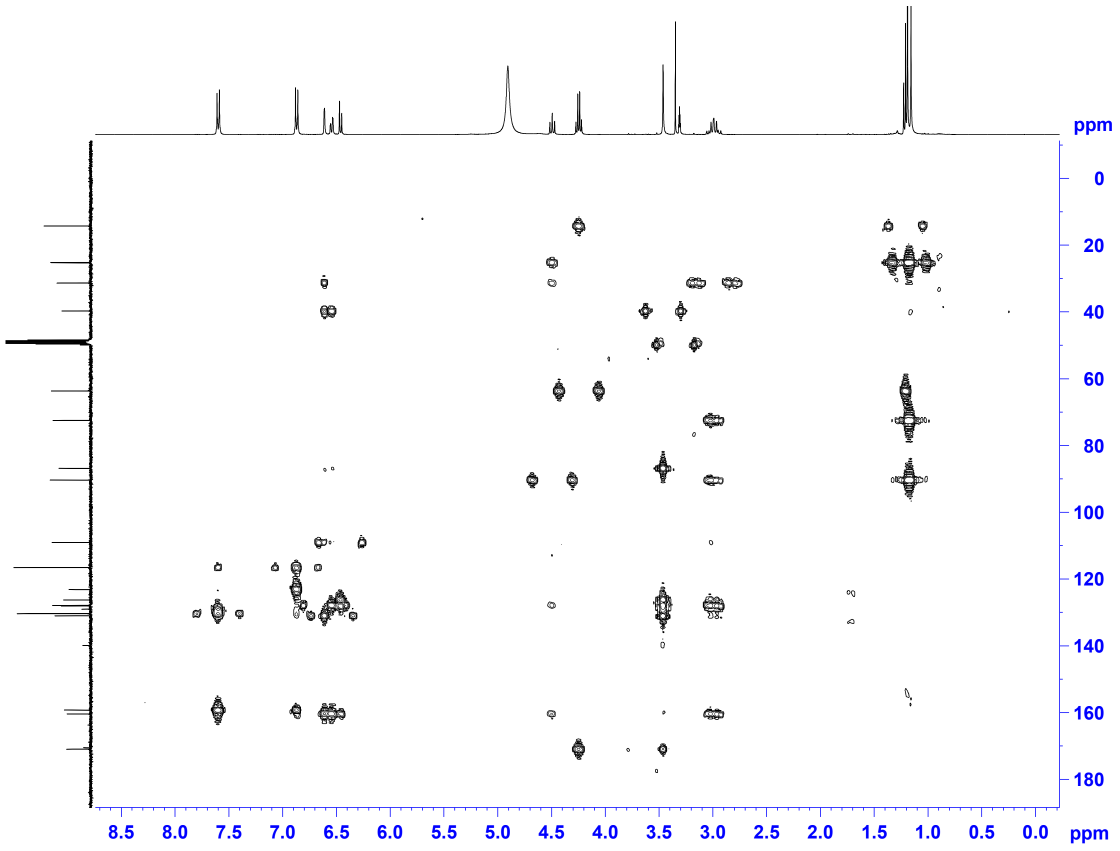

Figure S10 HMBC Spectrum of **2** in Methanol-d_4

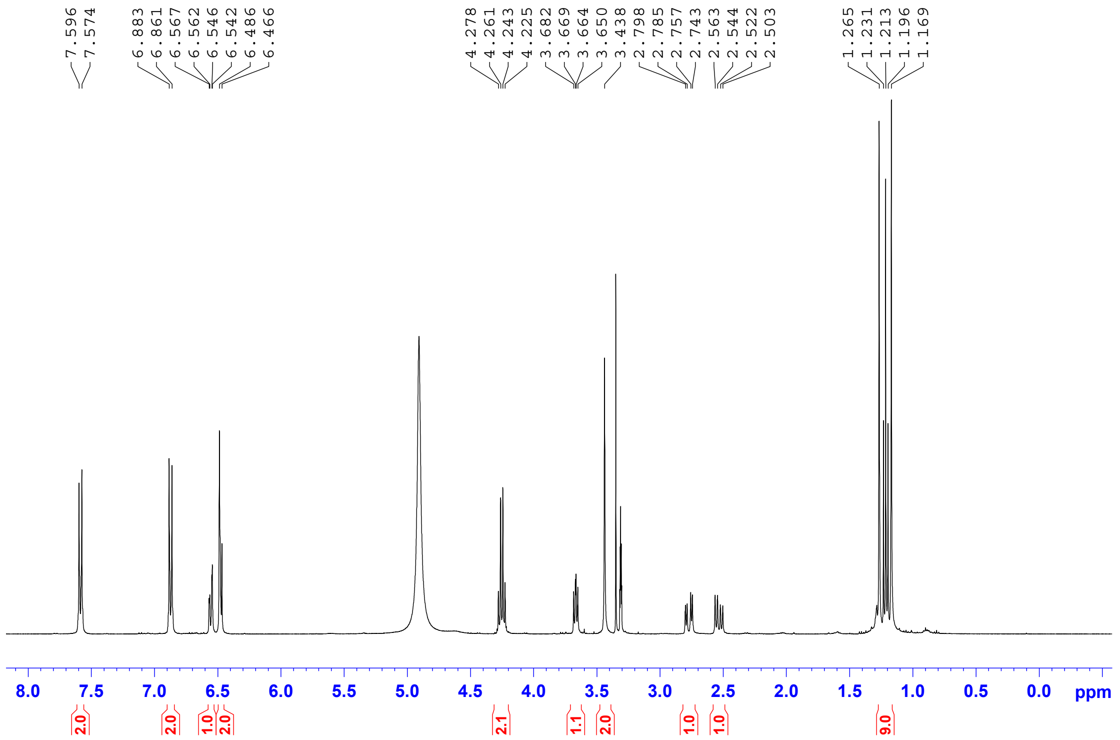

Figure S11 ^{1}H NMR Spectrum of **3** in Methanol-d_4 (400 MHz)

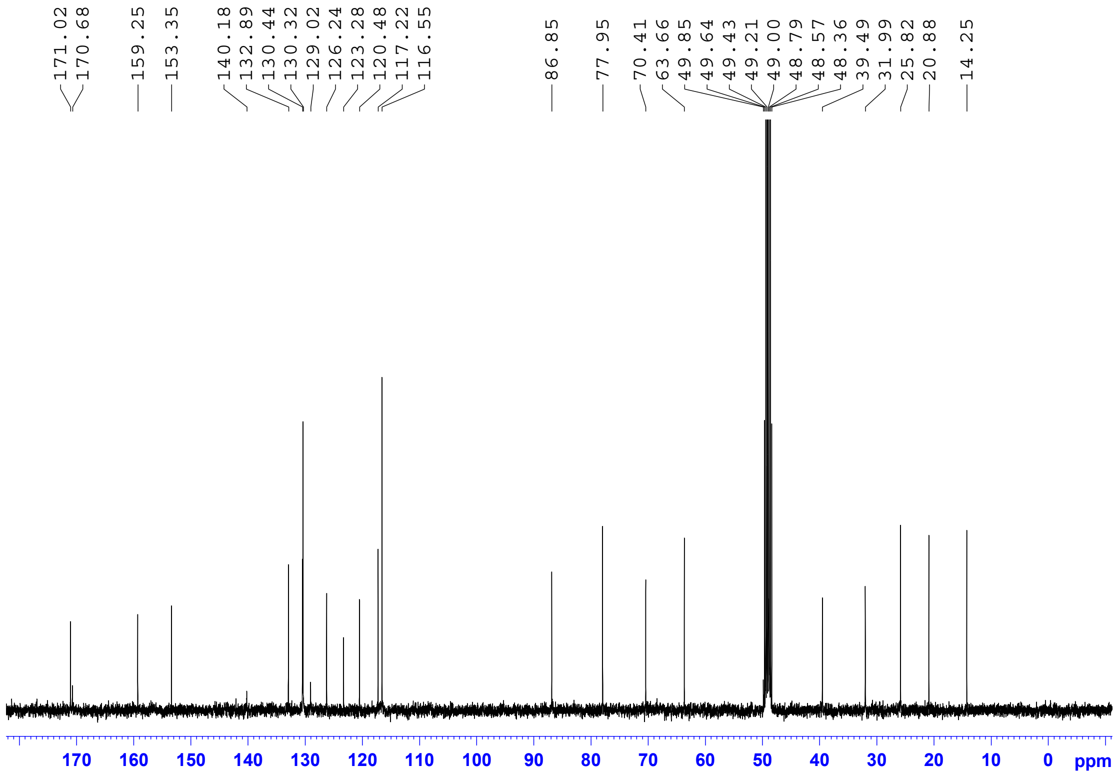

Figure S12 ^{13}C NMR Spectrum of **3** in Methanol-d_4 (100 MHz)

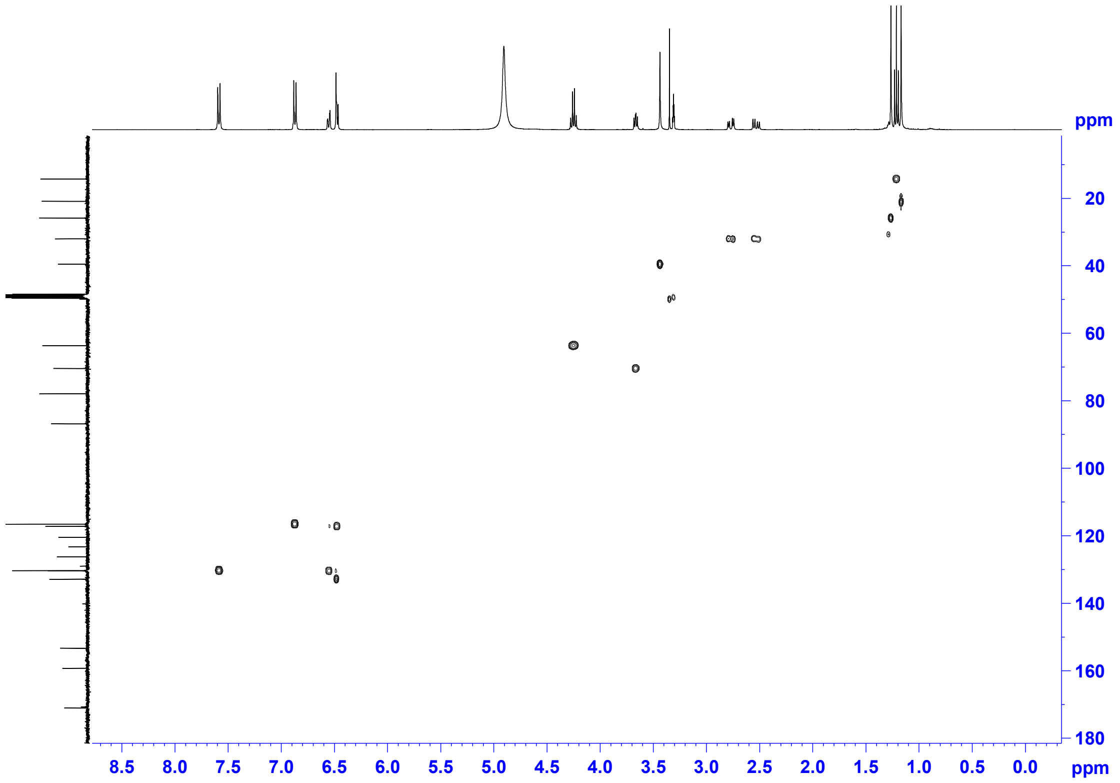

Figure S13 HSQC Spectrum of **3** in Methanol-d_4

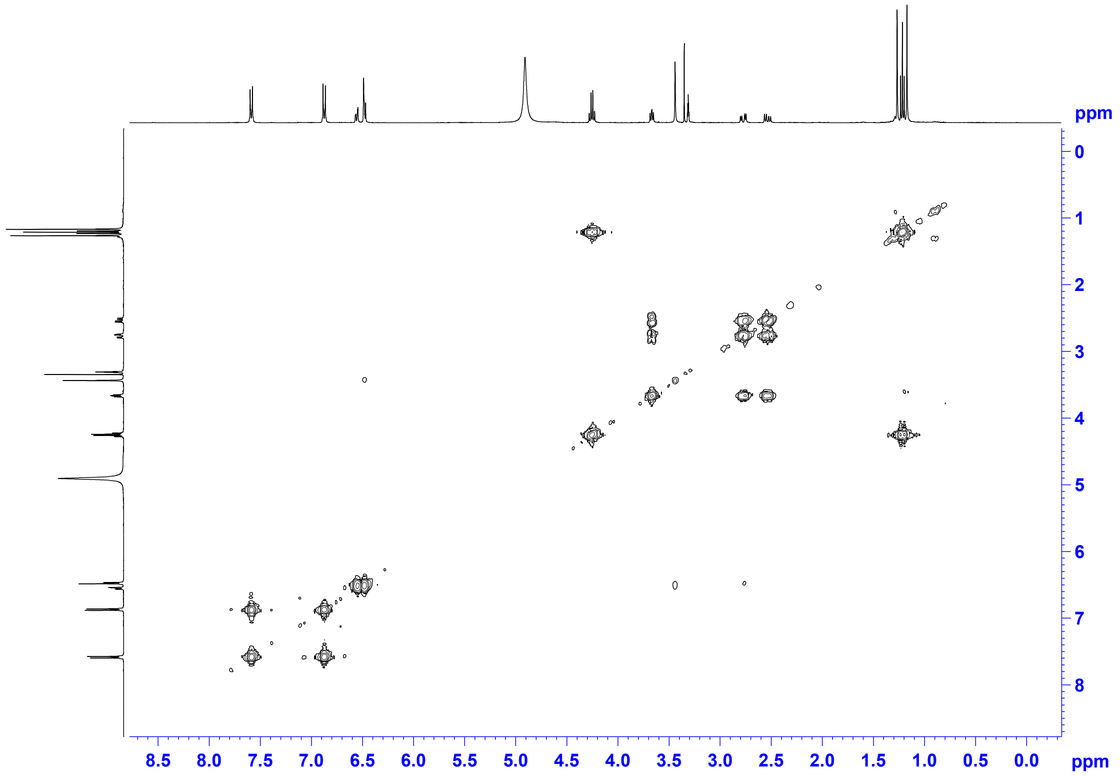

Figure S14 ^{1}H-^{1}H COSY Spectrum of **3** in Methanol-d_4

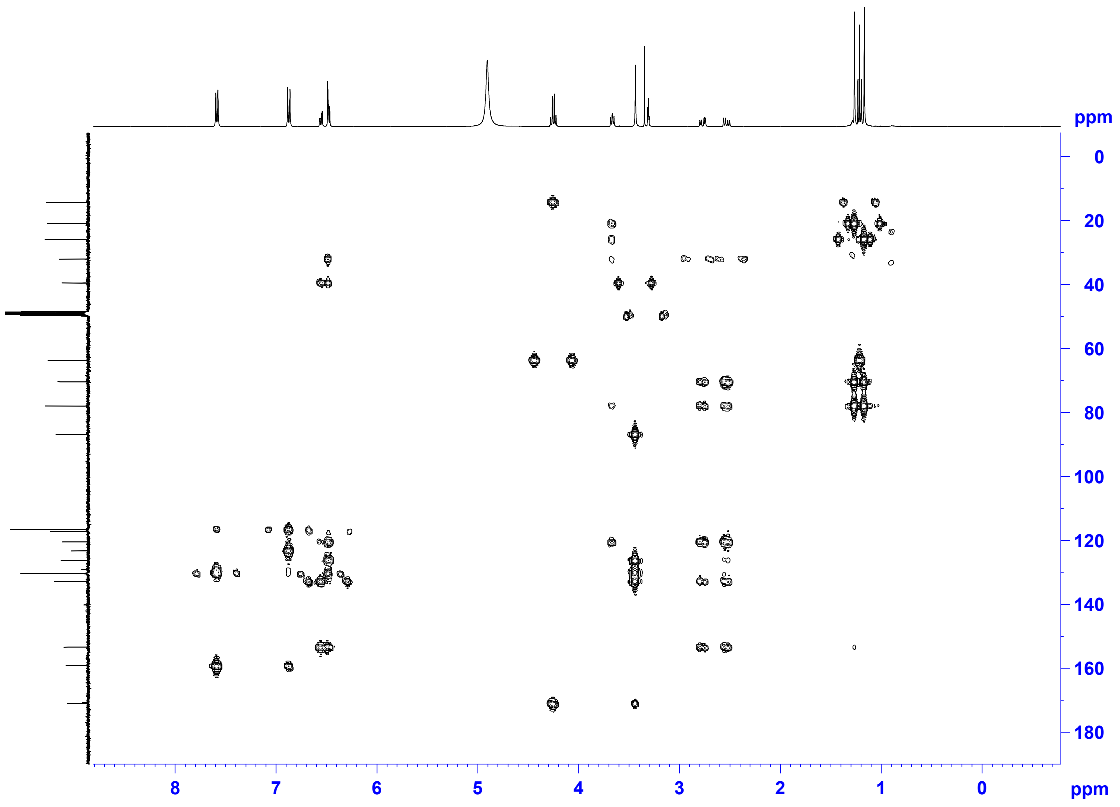

Figure S15 HMBC Spectrum of **3** in Methanol-d_4

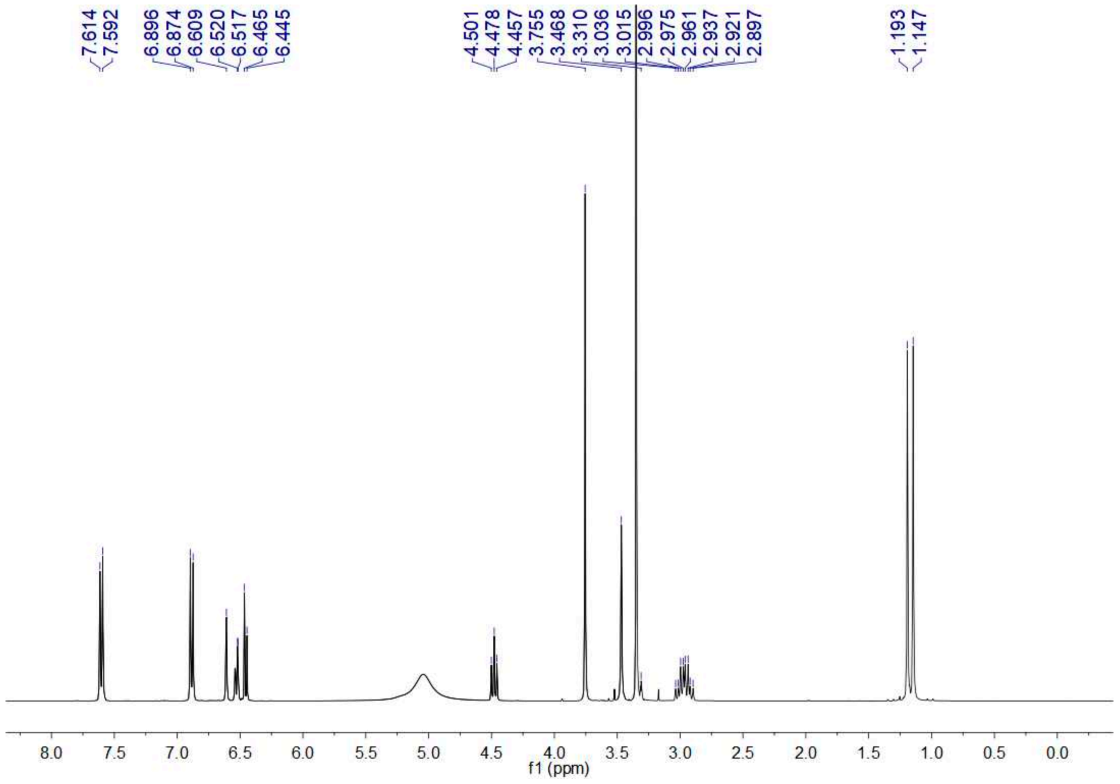

Figure S16 ^{1}H NMR Spectrum of **4** in Methanol-d_4 (400 MHz)

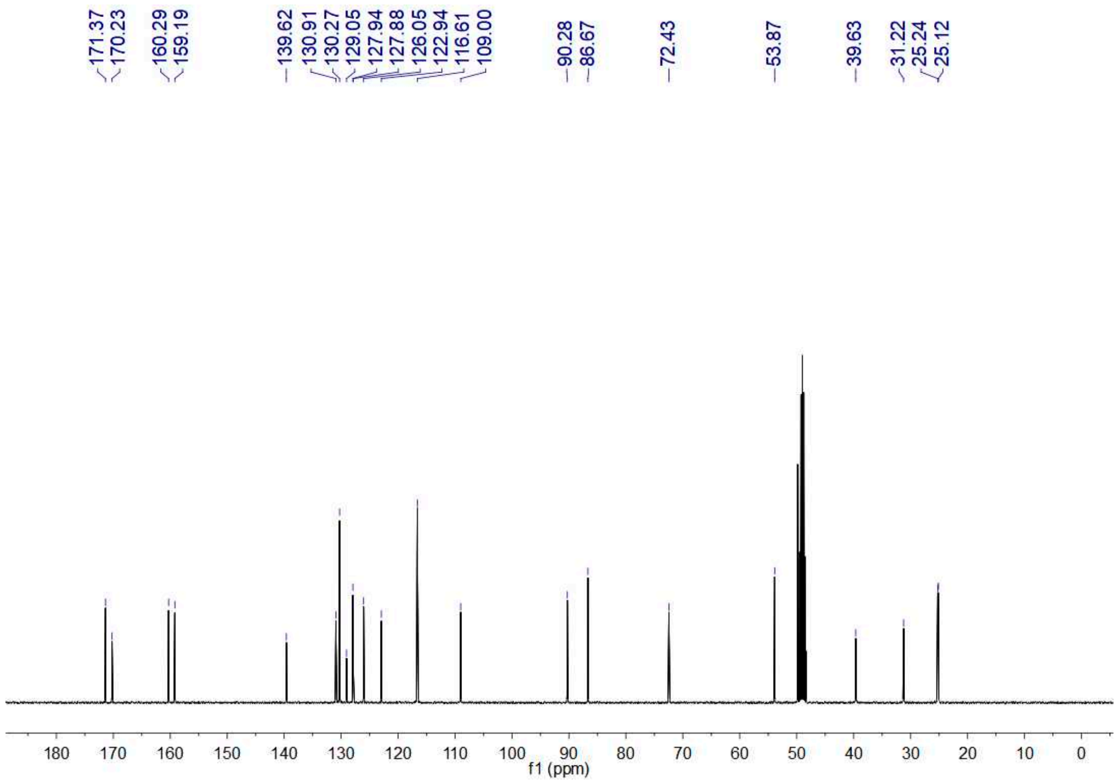

Figure S17 ^{13}C NMR Spectrum of **4** in Methanol-d_4 (100 MHz)

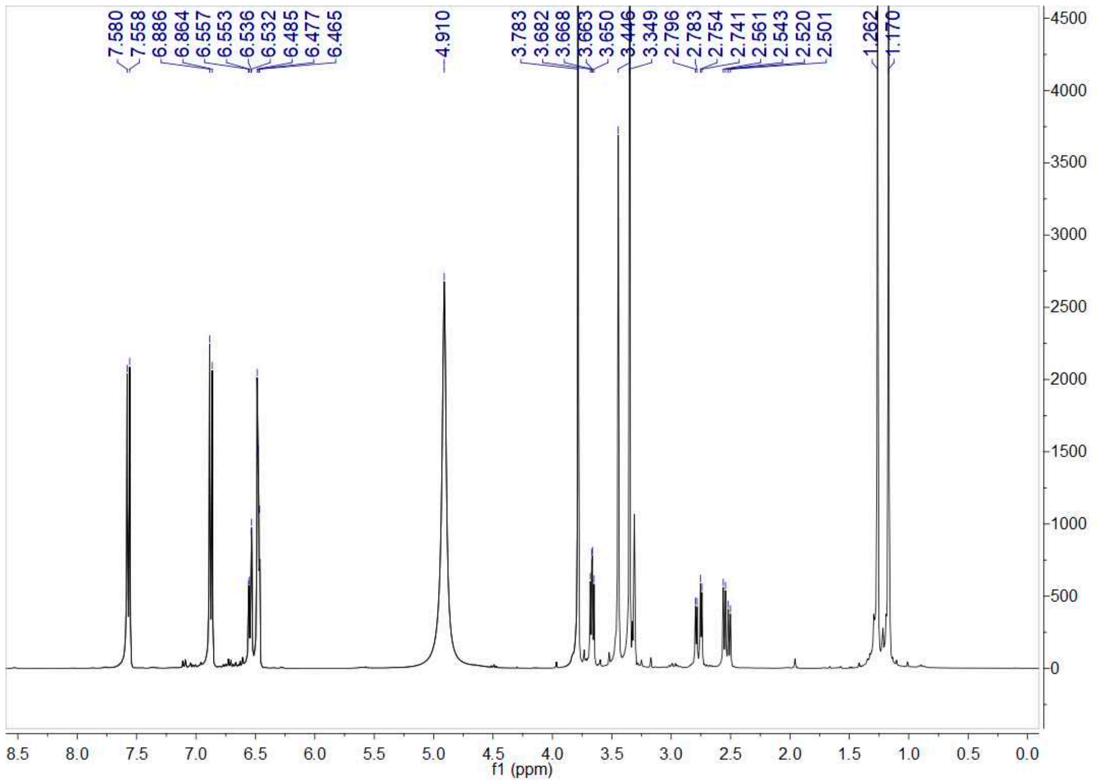

Figure S18 ^{1}H NMR Spectrum of **5** in Methanol-d_4 (400 MHz)

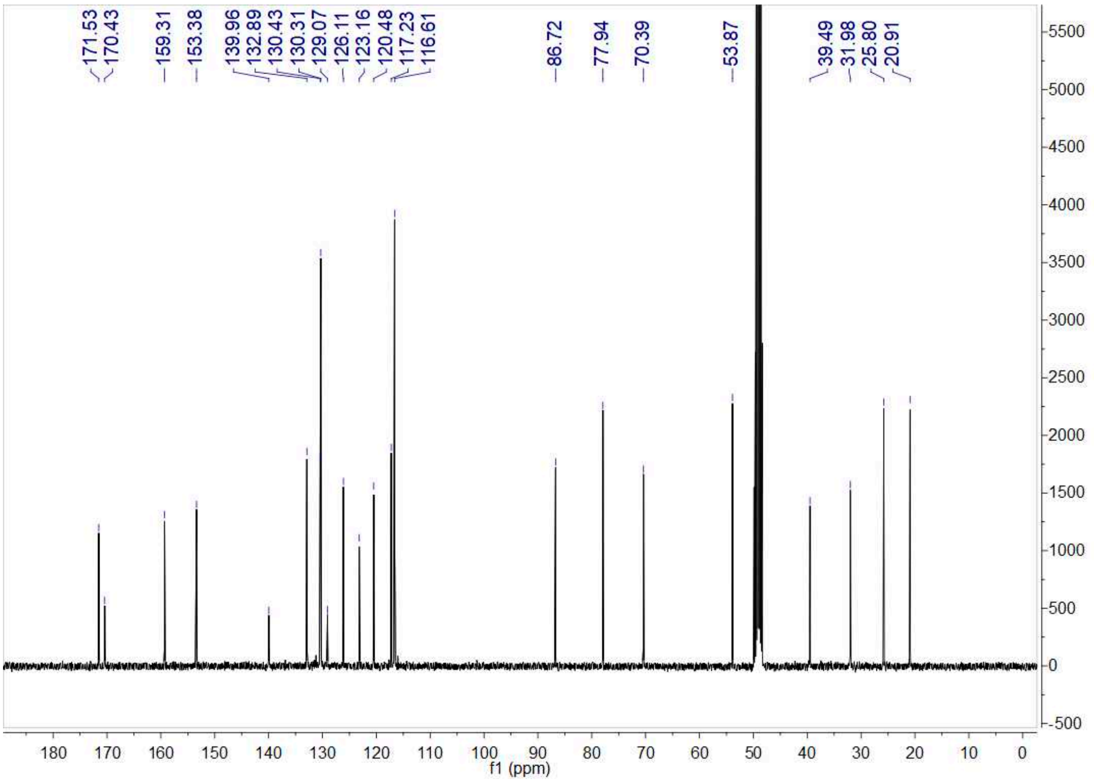

Figure S19 ^{13}C NMR Spectrum of **5** in Methanol-*d*4 (100 MHz)

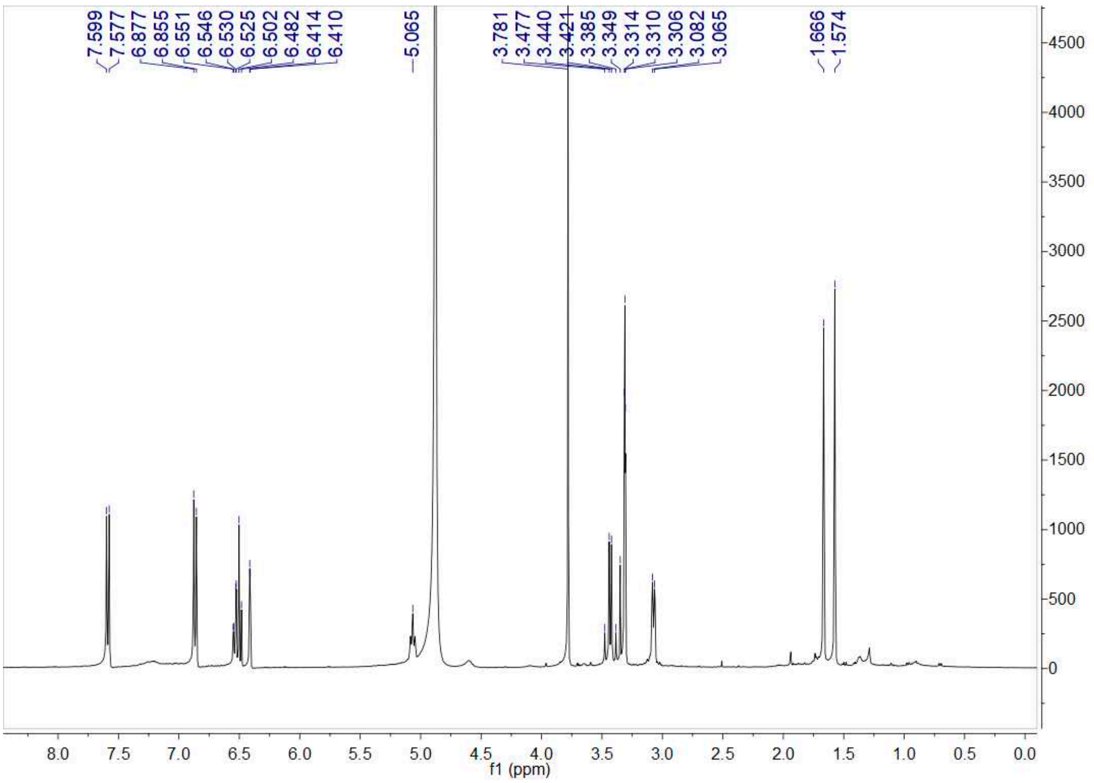

Figure S20 ^{1}H NMR Spectrum of **6** in Methanol-*d*4 (400 MHz)

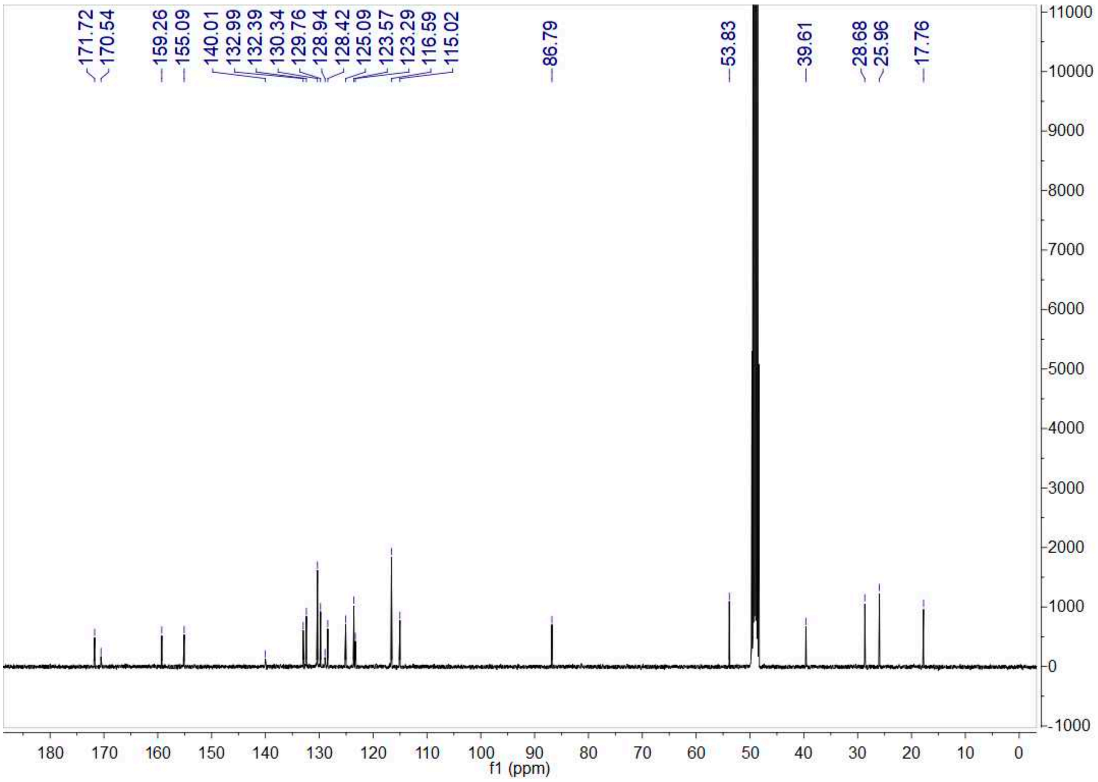

Figure S21 ^{13}C NMR Spectrum of **6** in Methanol-*d*4 (100 MHz)

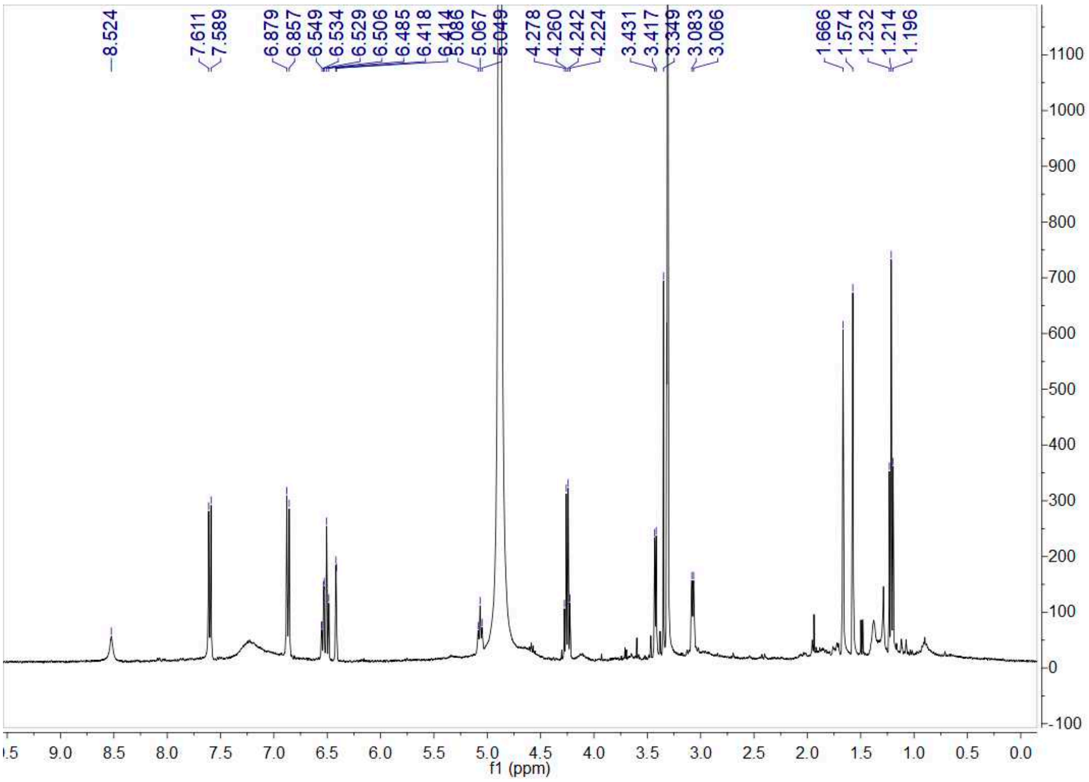

Figure S22 ^{1}H NMR Spectrum of **7** in Methanol-*d*4 (400 MHz)

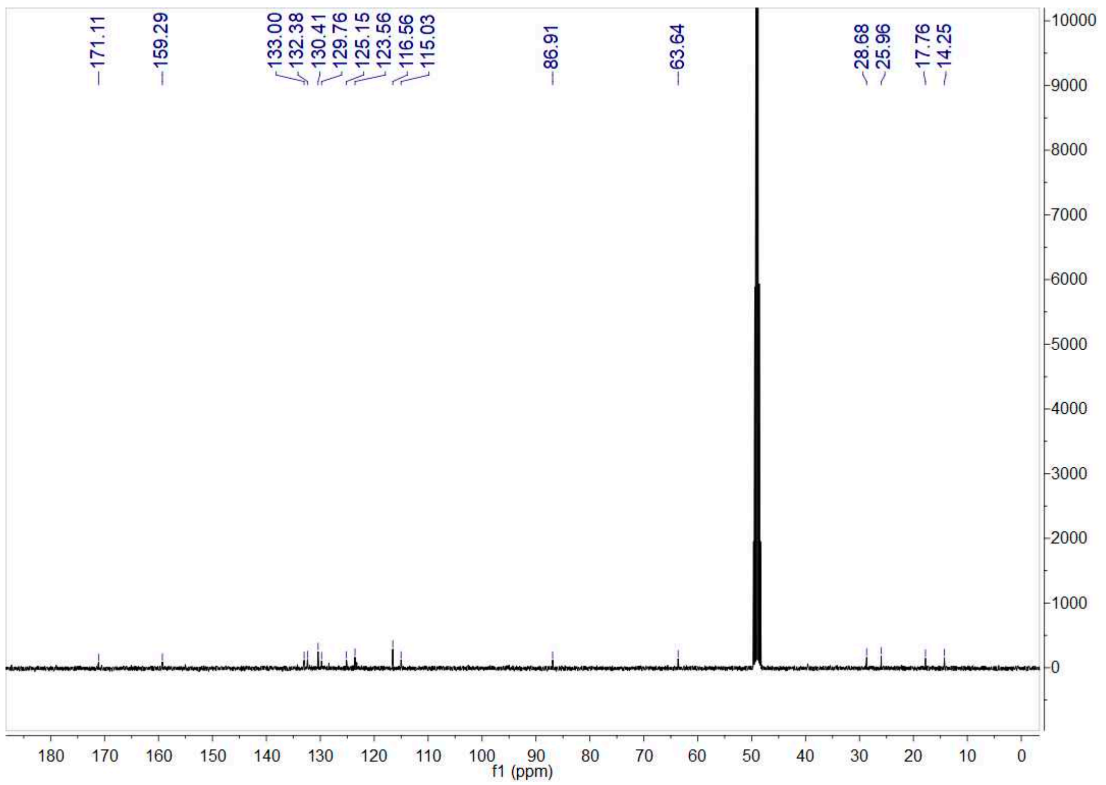

Figure S23 ^{13}C NMR Spectrum of **7** in Methanol-d_4 (100 MHz)

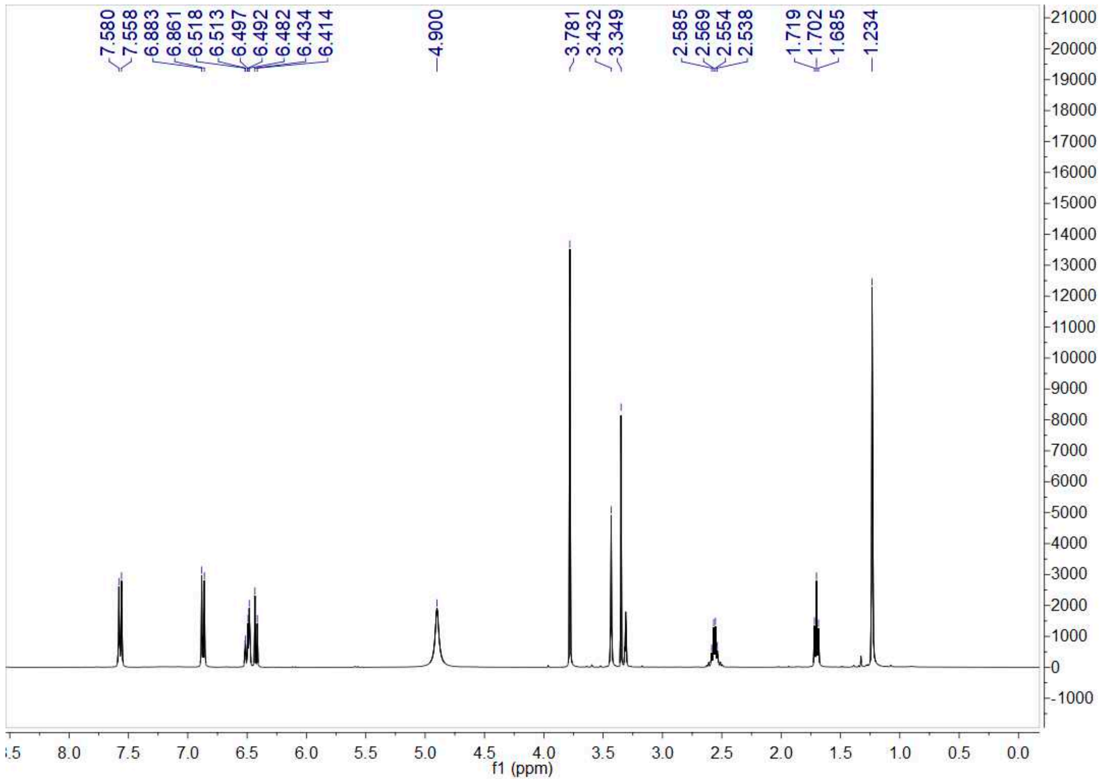

Figure S24 ^{1}H NMR Spectrum of **8** in Methanol-d_4 (400 MHz)

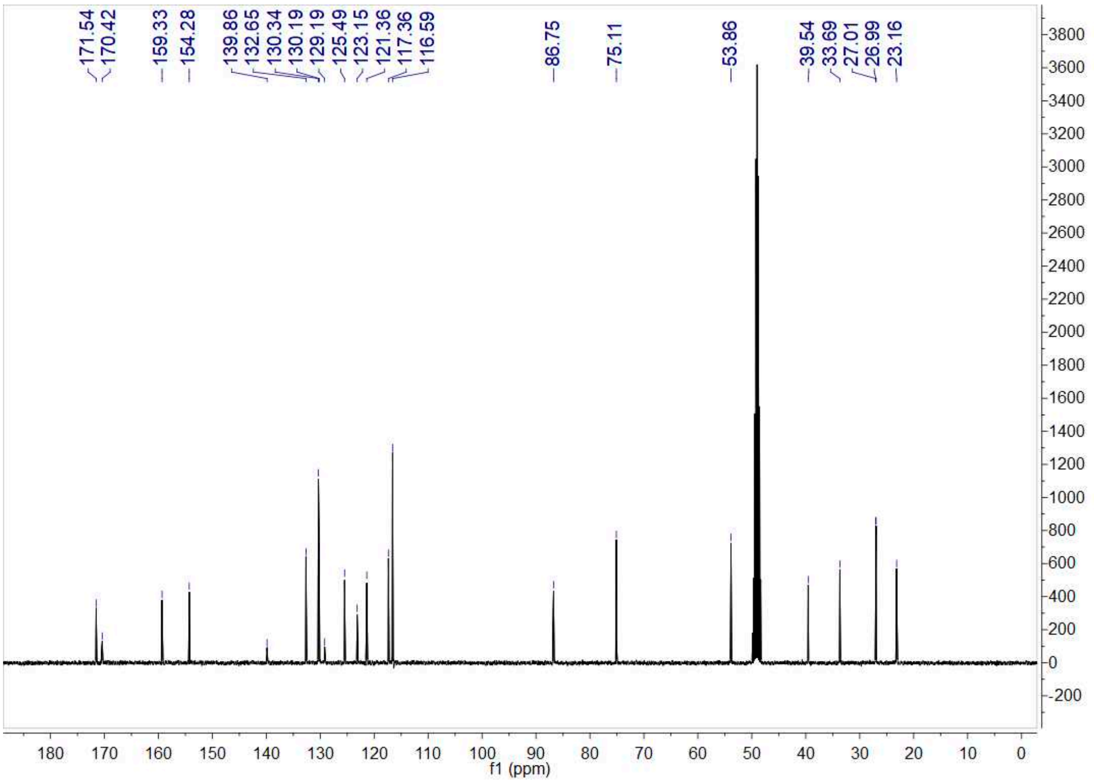

Figure S25 ^{13}C NMR Spectrum of **8** in Methanol-*d*4 (100 MHz)

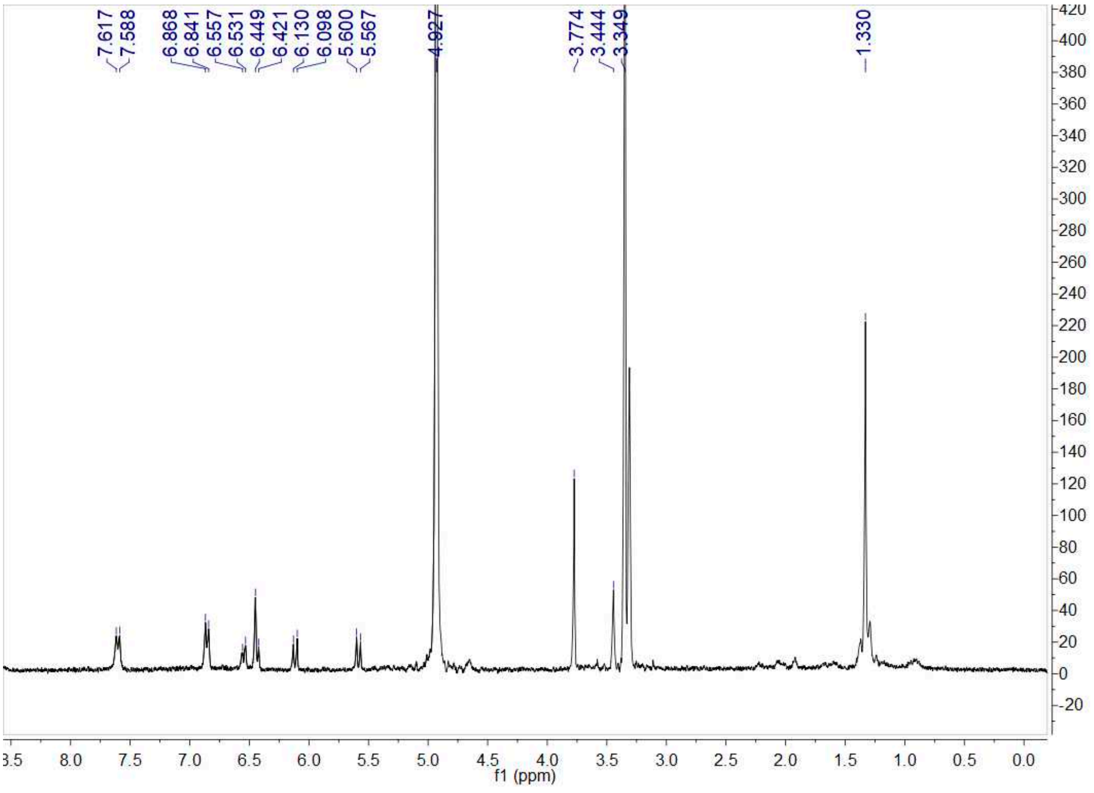

Figure S26 ^{1}H NMR Spectrum of **9** in Methanol-*d*4 (400 MHz)

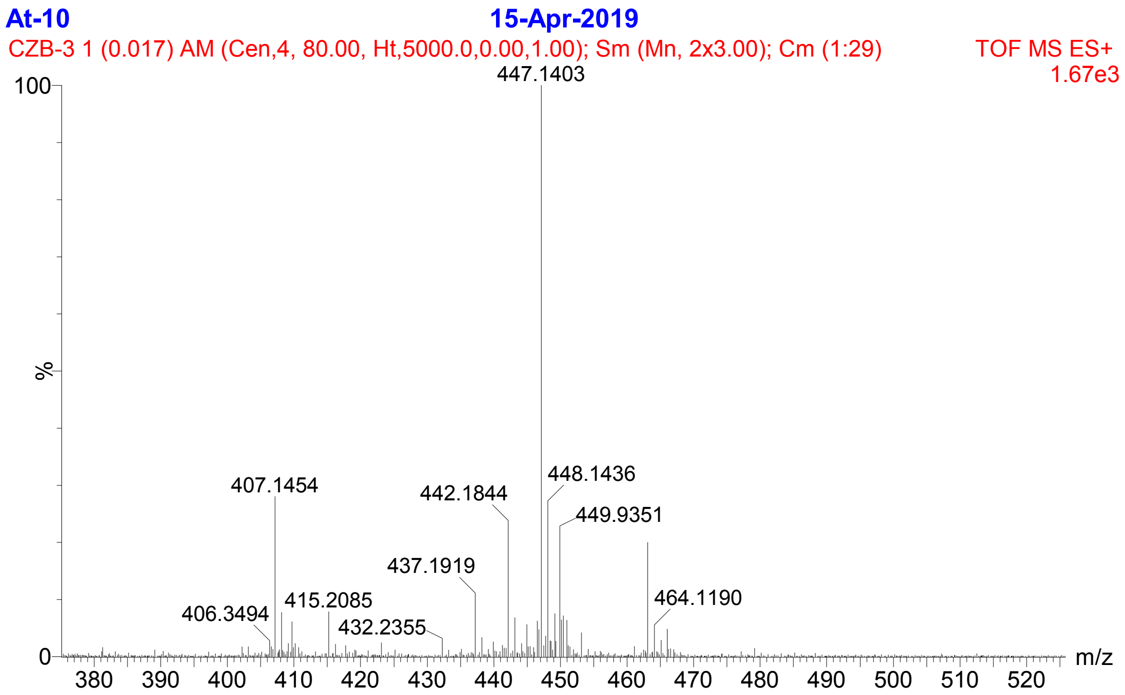

Figure S27 HRESIMS spectrum of **1**.

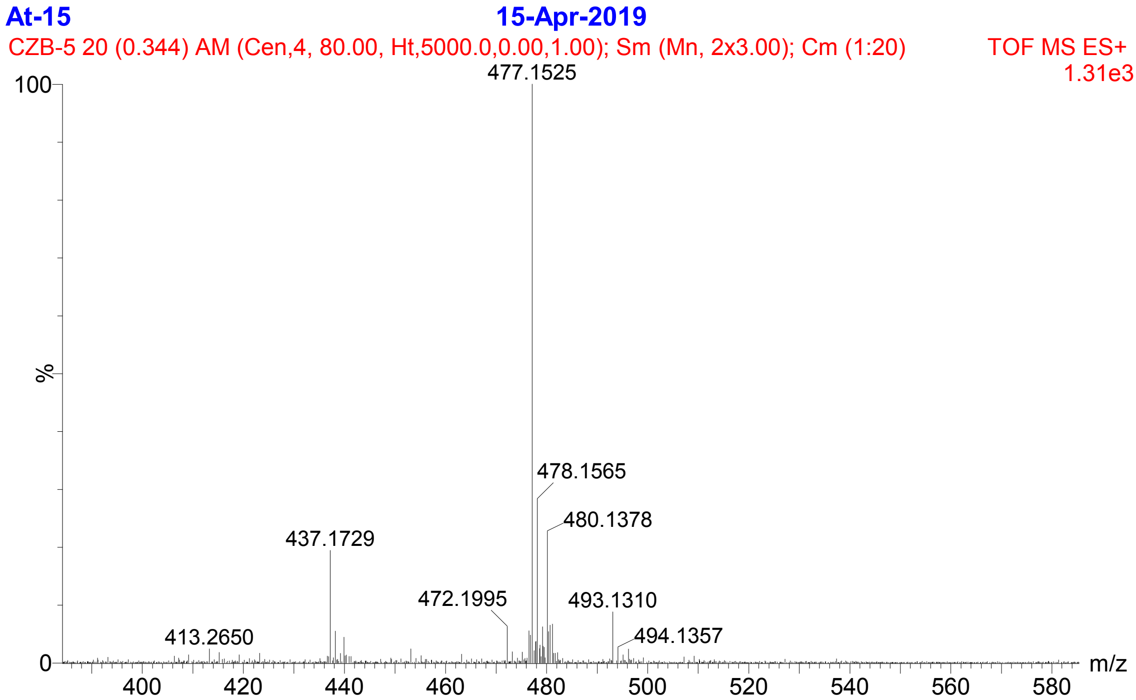

Figure S28 HRESIMS spectrum of **2**.

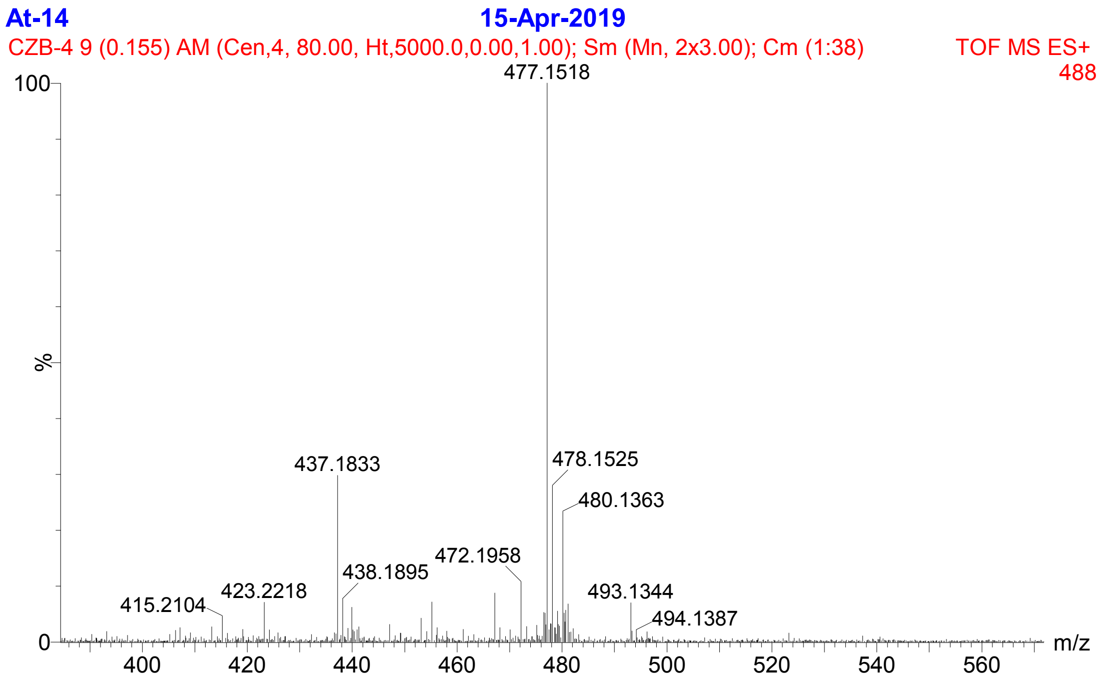

Figure S29 HRESIMS spectrum of **3**.
